# Investigation of the In Vitro and In Silico Anticancer Potential of Novel 4‐Arylthiazole‐Hydrazone Derivatives

**DOI:** 10.1111/cbdd.70387

**Published:** 2026-08-30

**Authors:** Zeynep Zişan Demirci, Erol Akgün, Gülşen Akalın Çiftçi, Leyla Yurttaş

**Affiliations:** ^1^ Department of Pharmaceutical Chemistry, Institute of Graduate Education Anadolu University Eskişehir Turkey; ^2^ Department of Pharmaceutical Chemistry, Faculty of Pharmacy Marmara University İstanbul Turkey; ^3^ Department of Biohemistry, Faculty of Pharmacy Anadolu University Eskişehir Turkey; ^4^ Department of Pharmaceutical Chemistry, Faculty of Pharmacy Anadolu University Eskişehir Turkey

**Keywords:** A549, apoptosis, caspase‐3 activation, cytotoxcity, DFT studies, hydrazone, MCF‐7, molecular dynamics simulation, moleuclar docking, thiazole

## Abstract

The anticancer efficacy of 4‐aryl‐2‐hydrazinothiazole derivatives, which combine a thiazole known for its wide range of medicinal applications and a hydrazide/hydrazone structure known for its unique biological properties, has been determined in numerous studies. In this study, novel 2‐[4‐[(2‐(4‐substituted thiazol‐2‐yl)hydrazono)methyl]phenoxy]acetic acid derivatives (**2a–l**) were synthesized and evaluated for their anticancer activity in MCF‐7 breast cancer, A549 lung cancer, and L929 normal cell lines using cytotoxicity, apoptosis, and caspase‐3 activation assays. The compounds were found to exhibit high cytotoxicity, particularly showing more selective and high‐potential antiproliferative activity on the MCF‐7 cell line. In apoptosis studies, compounds **2c**, **2d**, and **2h** caused programmed cell death in MCF‐7 cells exceeding 24%, close to cisplatin. Among these compounds, the derivative **2c** containing 4‐methoxyphenyl caused caspase‐3 activation at a level similar to cisplatin. To conduct in silico studies of the compounds, molecular docking studies with the (4QTX) caspase‐3 enzyme, molecular dynamics simulation for three compounds (**2d**, **2g** and **2l**), and Density Functional Theory (DFT) studies were performed. These studies have shown that the carboxylate groups in the compounds form strong and stable interactions with Arg64 and Arg207.

## Graphical abstract

The synthesized compounds exhibited excellent cytotoxicity on MCF‐7, breast cancer cell line with selective profile especially **2d** with IC_50_ value of 0.12 μM. Three compounds **2c**, **2d**, and **2h** caused almost as much apoptosis induction as cisplatin. Compound **2d** exhibited caspase‐3 activation at standard drug level. Molecular docking studies with the (4QTX) caspase‐3 enzyme and Density Functional Theory (DFT) studies were performed for all compounds. Molecular dynamics simulation for three compounds (**2d**, **2g**, and **2l**), and other studies showed that the carboxylate groups in the compounds form strong and stable interactions with Arg64 and Arg207.

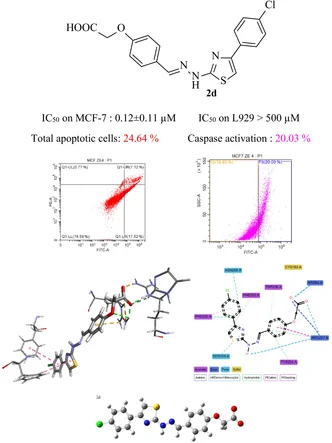

## 1 Introduction

As reported by the World Health Organization's (WHO) 2020 cancer analysis, cancer is the second leading cause of death globally (Sung et al. 2021). Cancer, which has high mortality rates, can be transmitted genetically, but it can also be caused by environmental factors such as smoking, alcohol use, or adverse weather conditions. According to a 2022 study by the International Agency for Cancer Development (IARC) and the American Cancer Society, nearly 19 million new cancer cases and 9.7 million cancer‐related deaths were reported worldwide. Among these globally diagnosed cancers, lung cancer was the most common, accounting for 12.4%, followed by breast cancer at 11.7% (Bray et al. 2024). Cancer chemotherapy, while involving a large number of different agents, has a very wide range of applications in treatment. However, the high toxic effects of the treatment necessitate ongoing research to discover new antitumor agents with high selectivity and low toxicity against cancer cells (Farghaly et al. 2020).

The synthesis of thiazoles, which have a 5‐membered heterocyclic structure, is of considerable importance today. In particular, there has been a significant increase in the number of thiazole‐containing drugs in the last decade, peaking in 2019 with the discovery of eight new drugs. Due to the electron pairs of their sulfur and nitrogen heteroatoms, they can interact with other molecules. This gives them the ability to bind to various biological targets (Lvov et al. 2018). The thiazole ring can exhibit biologically active properties in many diseases, primarily anticancer, antiviral, antibacterial, and antifungal. The thiazole ring is found in the structure of a number of well‐known anticancer drugs such as dabrafenib, dasatinib, and epotilones. Other effective drugs containing a thiazole ring include ritonavir (antiviral), abafungin (antifungal) (Nauen et al. 2003), and sulfathiazole (antibacterial) (Sharma et al. 2009; Carter et al. 1999; Oukoloff et al. 2019). Specifically regarding 2,4‐disubstituted thiazole derivatives, examples include thiazofurin and dasatinib, clinically used anticancer drugs that form the core structure of these molecules and offer a wide range of biological activity (Ayati et al. 2015).

Hydrazones are a class of agents that stand out due to their wide range of biological activity. When evaluated in terms of their anticancer activity, they are described as cytotoxic agents capable of preventing cellular progression in cancerous cells through various mechanisms. In special, 4‐arylthiazole‐hydrazone derivatives have demonstrated high anticancer activity against both lung and breast cancer cells in numerous studies (Wang et al. 2024; Evren et al. 2024; Tafere et al. 2025). Many thiazole‐based hydrazones and acylhydrazones (including benzothiazole/phenoxyacetyl analogs) carrying aromatic linkers exhibited micromolar‐level antiproliferative activity in A549 and MCF‐7 cells, confirming that this chemical structure is broadly cytotoxic against these cell lines. Furthermore, other studies have determined that this group of molecules acts by activating caspase‐3 (Evren et al. 2024; Kaya et al. 2025) and inducing apoptosis, in addition to many different anticancer pathways (Sever et al. 2025; Orujova et al. 2023). Building on this premise, current research synthesizes novel derivatives and evaluates their efficacy against various malignancies, specifically targeting MCF‐7 and A549 cell lines, investigating their cytotoxicity, caspase‐3 activation, and apoptosis induction, and comparing these experimental results with in silico studies.

## 2 Results and Discussion

### 2.1 Chemistry

**SCHEME 1:**
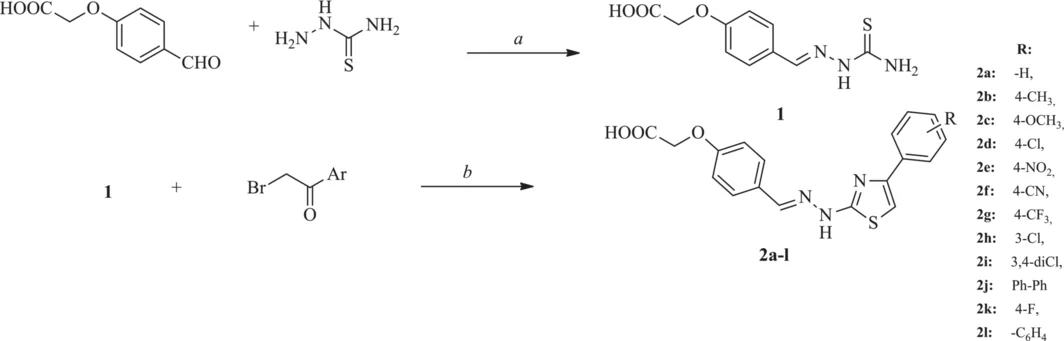
The synthetic protocol of the compounds (**2a–l**). Reactants and reagents: *a*: EtOH, reflux, 5 h; *b*: EtOH, reflux, 3 h.

Twelve new 2,4‐disubstituted thiazole derivatives (**2a–l**) were synthesized and their anticancer activities were evaluated. The compounds synthesized for this study were obtained using a two‐step synthesis method as shown Scheme 1. In the first step, based on the starting material, thiosemicarbazide and 4‐formylphenoxyacetic acid were used in a 1:1 M ratio and heated under reflux in ethanol. These two compounds formed a hydrazone bond (–C=N–NH–) via the condensation reaction between the aldehyde group and the nucleophilic –NH_2_ group of thiosemicarbazide. The resulting compound of step one, 2‐[4‐((2 carbamoylhydrazono)methyl)phenoxy]acetic acid (**1**), was reacted with equivalent amounts of phenacyl bromide derivatives in ethanol under reflux conditions. The reaction proceeds via condensation and dehydration, followed by ring closure to form thiazole‐containing derivatives. The reaction progress was monitored using thin‐layer chromatography (TLC). The structures and molecular compositions of the compounds (**2a–l**) were determined using ^1^H‐NMR, ^13^C‐NMR, IR, and mass spectrometry spectral and spectra of the final compounds were given in Supporting Information S1.

When the chemical structure of the final compounds (**2a–l**) was examined, they commonly contain phenylacetic acid residue and an azomethine group. In the ^1^H‐NMR spectra of the compounds, methylene protons linked to the carboxylic acid group were observed between 4.73 and 4.69 ppm. The proton at the C5 proton of the thiazole ring was resonated as singlet peak between 7.13 and 7.38 ppm whereas amino protons were appeared in the range of 12.03–12.42 ppm in all derivatives. The azomethine proton was also observed at about 7.97–8.05 ppm. While aromatic protons are recorded in the range of 6.69–8.28 ppm, the signals of phenoxy protons belonging to these derivatives have been detected in the range of 6.69–7.79 ppm. In the ^13^C‐NMR spectra of the compounds, signals for the aromatic carbons were observed in the range of 115.35–141.93 ppm. A characteristic signal for thiazole ring C2 carbon appeared in the range of 159.14–159.31 ppm. Signals corresponding to the carbonyl and α‐methylene carbons showed a signal at 168.95–170.54 ppm and 64.90–64.97 ppm, respectively. In the FT‐IR spectra, the characteristic strong absorption band for the carbonyl group (C=O) was observed between 1634 and 1767 cm^−1^ in all derivatives. The aliphatic C‐H stretching vibrations appeared in the region of 2988–2839 cm^−1^. The target compound were further supported by the absorption band for the OH and NH stretching vibrations in the region of 3647–3663 cm^−1^ and 3130–3275 cm^−1^, respectively. According to the mass spectra of the compounds, M + 1 peaks consistent with their molecular weights were obtained. Only one isomer was obtained during the synthesis of the compounds, and it was determined according to the literature that this isomer was *E* (Takrouri et al. 2014).

### 2.2 Biological Evaluation

#### 2.2.1 Antiproliferative Activity

The **2a–l** compounds were tested on A549, MCF‐7, and L929 cell lines. The half maximal inhibition concentration (IC_50_) was calculated for all compounds and the results were given in Table 1. The efficacy of each compound was quantified as the inhibitory concentration against the respective cell lines, with all values expressed in μg/mL.

**TABLE 1:** IC_50_ values (μM) of the compounds against MCF‐7, A549, and L929 cell lines (μM).

Compounds	A549	MCF‐7	L929
**2a**	52.44 ± 5.84	76.39 ± 26.97	44.48 ± 8.15
**2b**	46.27 ± 4.19	11.37 ± 6.14	37.14 ± 1.71
**2c**	33.54 ± 4.66	8.27 ± 0.84	43.75 ± 1.31
**2d**	> 500	**0.12 ± 0.11**	> 500
**2e**	35.15 ± 2.87	13.67 ± 0.49	45.19 ± 0.94
**2f**	34.24 ± 4.27	17.58 ± 4.41	60.23 ± 10.99
**2g**	> 500	17.97 ± 4.49	> 500
**2h**	71.14 ± 8.82	4.74 ± 0.22	32.37 ± 5.09
**2i**	> 500	**0.39 ± 0.21**	43.75 ± 1.31
**2j**	> 500	3.10 ± 0.66	67.17 ± 17.56
**2k**	110.79 ± 28.28	1.96 ± 0.30	56.77 ± 12.62
**2l**	216.82 ± 0.31	1.45 ± 0.26	128.37 ± 40.96
**Cisplatin**	43.61 ± 1.05	13.40 ± 2.86	—

*Note:* —, not tested. Bold values show higher activity.

The cytotoxic potential of the 12 synthesized compounds was ascertained against A549, MCF‐7, and L929 cell lines by means of the MTT assay. Cisplatin was employed as the positive control for comparison of the results. This colorimetric method relies on the enzymatic transformation of the yellow tetrazolium salt (3‐(4,5‐dimethylthiazol‐2‐yl)‐2,5‐diphenyltetrazolium bromide) into insoluble purple formazan crystals, a process catalyzed by various mitochondrial enzymes, principally succinate dehydrogenase, in metabolically competent cells. Consequently, the magnitude of the formazan signal provides a reliable measure of cellular viability. Following the dissolution of the resulting formazan crystals in DMSO, the optical density at 570 nm was recorded by spectrophotometric analysis (Yurttaş et al. 2021). Based on the IC_50_ values obtained from the calculations, it was reported that compounds **2c** (33.54 ± 4.66 μM), **2e** (35.15 ± 2.87 μM), and **2f** (34.24 ± 4.27 μM) yielded results close to cisplatin (43.61 ± 1.05) on A549 cells and also higher potency on L929 cell lines which means lower selectivity (SI ≈ 1.5). Compounds **2a** and **2b** exhibited potency similar to cisplatin but were found to be cytotoxic to L929. Compounds **2d**, **2g**, **2i**, and **2j** did not show cytotoxicity to A549 even at the highest dose tested (> 500 μM). In cell experiments conducted on the MCF‐7 cell line, compounds **2d** (0.12 ± 0.11 μM) and **2i** (0.39 ± 0.21 μM) showed perfect inhibitory effect compared to cisplatin (13.40 ± 2.86 μM). In addition, compounds **2b** (11.37 ± 6.14), **2c** (8.27 ± 0.84), **2h** (4.74 ± 0.22), **2j** (3.1 ± 0.66), **2k** (1.96 ± 0.30), and **2l** (1.45 ± 0.26) with IC_50_ demonstrated antiproliferative activity against MCF‐7 cells, showing higher efficacy than cisplatin and exhibiting a non‐toxic profile. Furthermore, compounds **2e**, **2f**, and **2g** also showed selective cytotoxicity close to the standard drug. Of the compounds, only **2a** failed to show selectivity against MCF‐7 and was found to be weakly cytotoxic. The compounds were found to exhibit significant potency and selectivity against the MCF‐7 cell line, but not to the desired extent against A549 cells.

A preliminary structure–activity relationship was evident from the antiproliferative data. In MCF‐7 cells, replacement of the unsubstituted phenyl group of compound **2a** with a para‐chloro substituent in compound **2d** markedly increased antiproliferative activity, while the 3,4‐dichloro analogue **2i** also exhibited one of the lowest IC_50_ values in the series. The lower activities of the meta‐chloro derivative **2h** and the para‐trifluoromethyl derivative **2g** indicate that electron‐withdrawing character or lipophilicity alone cannot explain the observed activity. The position, size, and spatial orientation of the substituent also appear to be important. The biphenyl derivative **2j**, the 4‐fluoro derivative **2k**, and the naphthyl derivative **2l** retained low‐micromolar activity against MCF‐7 cells, suggesting that selected hydrophobic and extended aromatic groups may favor antiproliferative activity in this cell line.

A different structure–activity pattern was observed in A549 cells. The 4‐methoxy derivative **2c**, the 4‐nitro derivative **2e**, and the 4‐cyano derivative **2f** showed the lowest IC_50_ values, whereas compounds **2d**, **2g**, **2i**, and **2j** did not reach 50% growth inhibition at the highest concentration tested. These findings indicate that substitution on the 4‐arylthiazole moiety is a major determinant of cell‐line preference. The observed cell‐line selectivity may therefore also reflect differences associated with the variable aryl substituent, physicochemical properties, cellular uptake, and interactions with additional intracellular targets.

#### 2.2.2 Apoptosis Induction

Cancer causes various mutations in the cell DNA, leading cells to continue reproducing without interruption. These malignant cells undergo various adaptations, making them impossible to eliminate through intracellular control systems. The most important of these control systems is the apoptosis mechanism (Pandya et al. 2023). A deficiency in the apoptosis mechanism results in the survival and proliferation of malignant cells (Moslehi et al. 2023). The death of a cell through inflammation that also affects surrounding tissues is called necrosis. Conversly, in cell culture experiments, cancer cells are expected to die not through necrosis, but through apoptosis, which is a planned cell death (Elmore 2007).

Double staining‐based flow cytometry is a fundamental method used to quantitatively determine the effects of tested compounds on apoptosis under in vitro conditions. Phosphatidylserine, located in the inner part of the cell membrane, is transported to the outer surface of the membrane in the early apoptosis phase. Annexin‐V FITC binds to the lipid transported to the outer surface. Propidium iodide (PI) stain penetrates cells that have lost membrane integrity, thus allowing PI to differentiate between late apoptosis and necrotic cell populations (Cheng et al. 2015).

**FIGURE 1:**
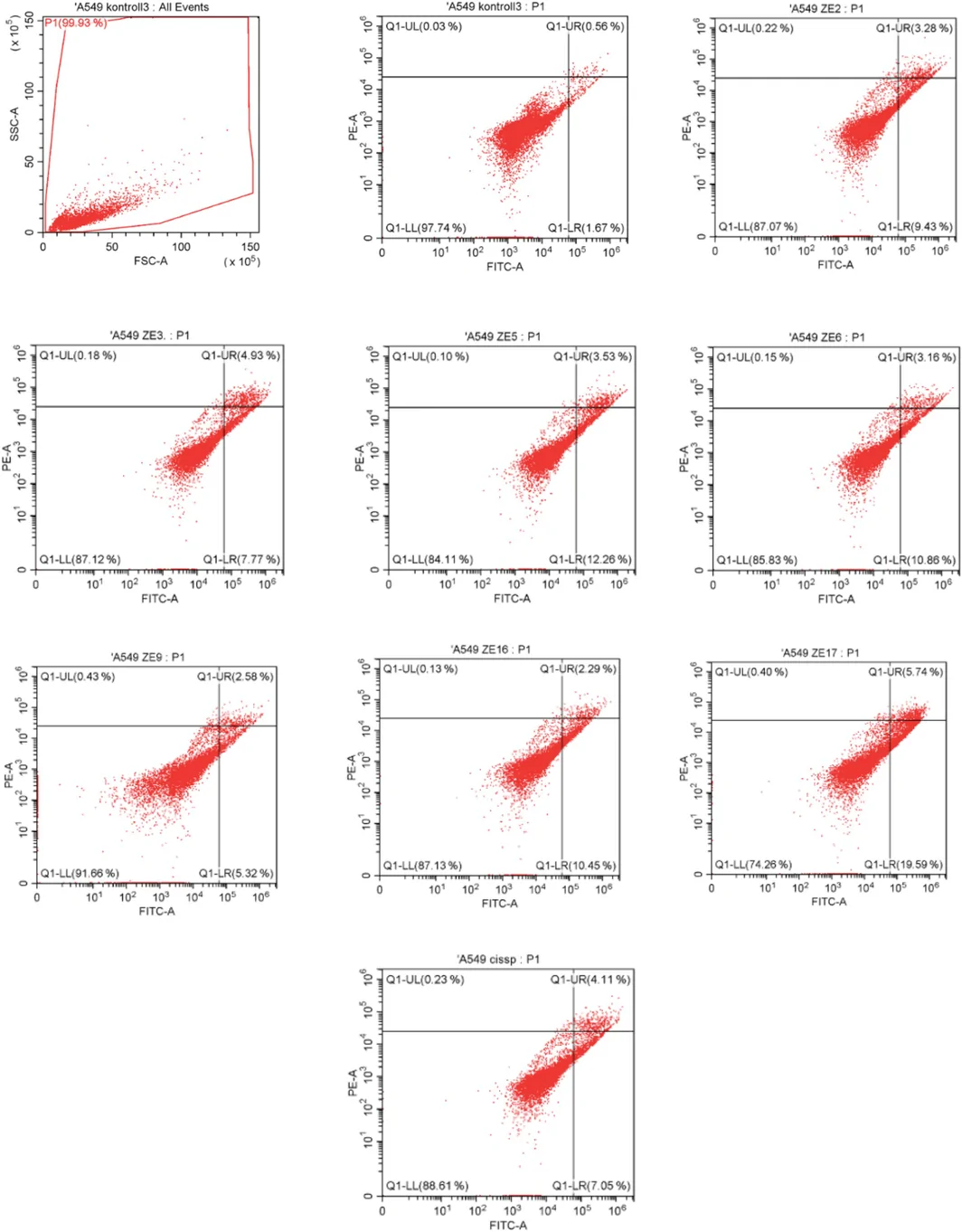
Annexin‐V FITC/PI diagram for A549 cell line in **control 1**, **control 2**, and treated with **2b**, **2c**, **2e**, **2f**, **2h**, **2k**, **2l** compounds and **cisplatin** respectively.

Compound **2l** exhibited the highest apoptosis induction on the A549 cell line, markedly outperforming cisplatin with an inhibition rate of 25.33%. Furthermore, compounds **2c** (12.70), **2e** (15.79), **2f** (14.02), and **2k** (12.74) displayed potency comparable to the reference drug cisplatin (11.16); these values are summarized in Table 2. The diagrams acquired from flow cytometry on the A549 cell line were also represented in Figure 1.

**TABLE 2:** Annexin‐V FITC/PI flow cytometry results for compounds **2a–l** and cisplatin in A549 cells.

Compound	Viable (LL)	Necrotic (UL)	Late apoptotic (UR)	Early apoptotic (LR)	Total apoptotic (UR + LR)
**2b**	87.07	0.22	3.28	9.43	9.65
**2c**	87.12	0.18	4.93	7.77	**12.70**
**2e**	84.11	0.10	3.53	12.26	**15.79**
**2f**	85.83	0.15	3.16	10.86	**14.02**
**2h**	91.66	0.40	2.58	5.32	7.9
**2k**	87.13	0.13	2.29	10.45	**12.74**
**2l**	74.26	0.40	5.74	19.59	**25.33**
**Cisplatin**	88.61	0.23	4.11	7.05	11.16
**Control**	97.74	0.03	0.56	1.67	2.23

*Note:* Bold values show higher activity.

**FIGURE 2:**
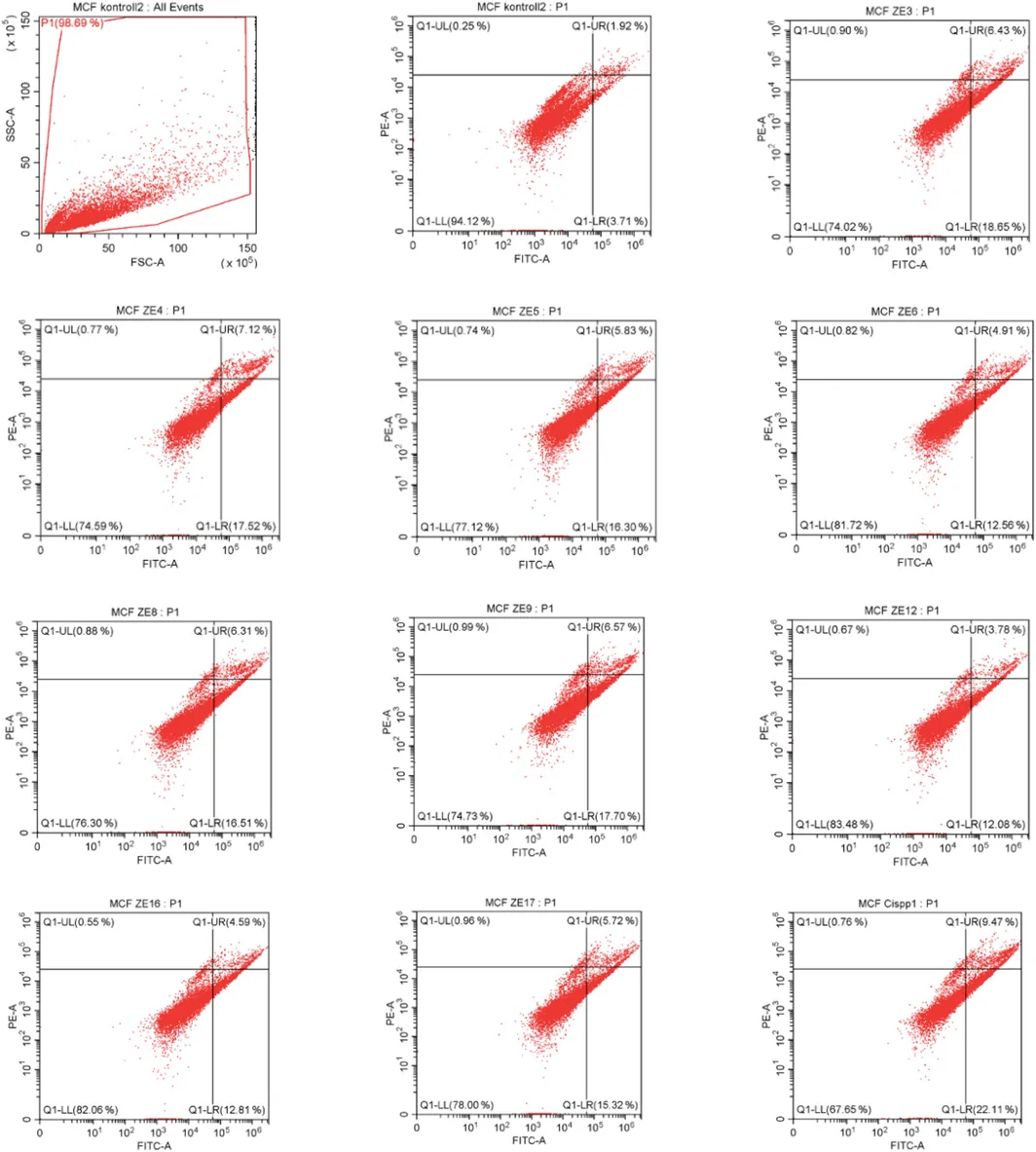
Annexin‐V FITC/PI diagram for MCF‐7 cell line in **control 1**, **control 2**, and treated with **2c**, **2d**, **2e**, **2f**, **2g**, **2h**, **2i**, **2k**, **2l** compounds and **cisplatin** respectively.

Regarding the Annexin‐V results observed in MCF‐7 cells, the reference drug cisplatin showed the highest apoptosis percentage at 31.58%. Compounds **2c** (25.08%), **2d** (24.64%), and **2h** (24.27%) demonstrated apoptotic activities comparable to cisplatin by inducing apoptosis in approximately one‐quarter of the cells (Table 3). In addition, compounds **2e**, **2g**, and **2l** also caused apoptosis in more than 20% of cases. The flow cytometry quadrants on MCF‐7 cell line were given in Figure 2.

**TABLE 3:** Annexin‐V FITC/PI flow cytometry results for compounds **2a–l** and cisplatin in MCF‐7 cells.

Compound	Viable (LL)	Necrotic (UL)	Late apoptotic (UR)	Early apoptotic (LR)	Total apoptotic (UR + LR)
**2c**	74.02	0.90	6.43	18.65	**25.08**
**2d**	74.59	0.77	7.12	17.52	**24.64**
**2e**	77.12	0.74	5.83	16.30	22.13
**2f**	81.72	0.82	4.91	12.56	17.47
**2g**	76.30	0.88	6.31	16.51	22.82
**2h**	74.73	0.99	6.57	17.70	**24.27**
**2i**	83.48	0.67	3.78	12.08	15.86
**2k**	82.06	0.55	4.59	12.81	17.40
**2l**	78.00	0.96	5.72	15.32	21.04
**Cisplatin**	67.65	0.76	9.47	22.11	**31.58**
**Control**	94.12	0.25	1.92	3.71	5.63

*Note:* Bold values show higher activity.

#### 2.2.3 Caspase‐3 Activation

Caspase‐3 is an enzyme involved in both the intrinsic and extrinsic pathways of apoptosis, or programmed cell death. While initiator caspases trigger the process, it is caspase‐3 that acts as the “executioner” protease, causing both biochemical and morphological breakdown of the cell. Following enzyme activation, DNA repair mechanisms cease, leading to the destruction of the cytoskeleton. This destruction is not necrosis‐like disintegration; on the contrary, rather a process in which the cell is eliminated through apoptosis without damaging neighboring tissues (Porter and Jänicke 1999).

**FIGURE 3:**
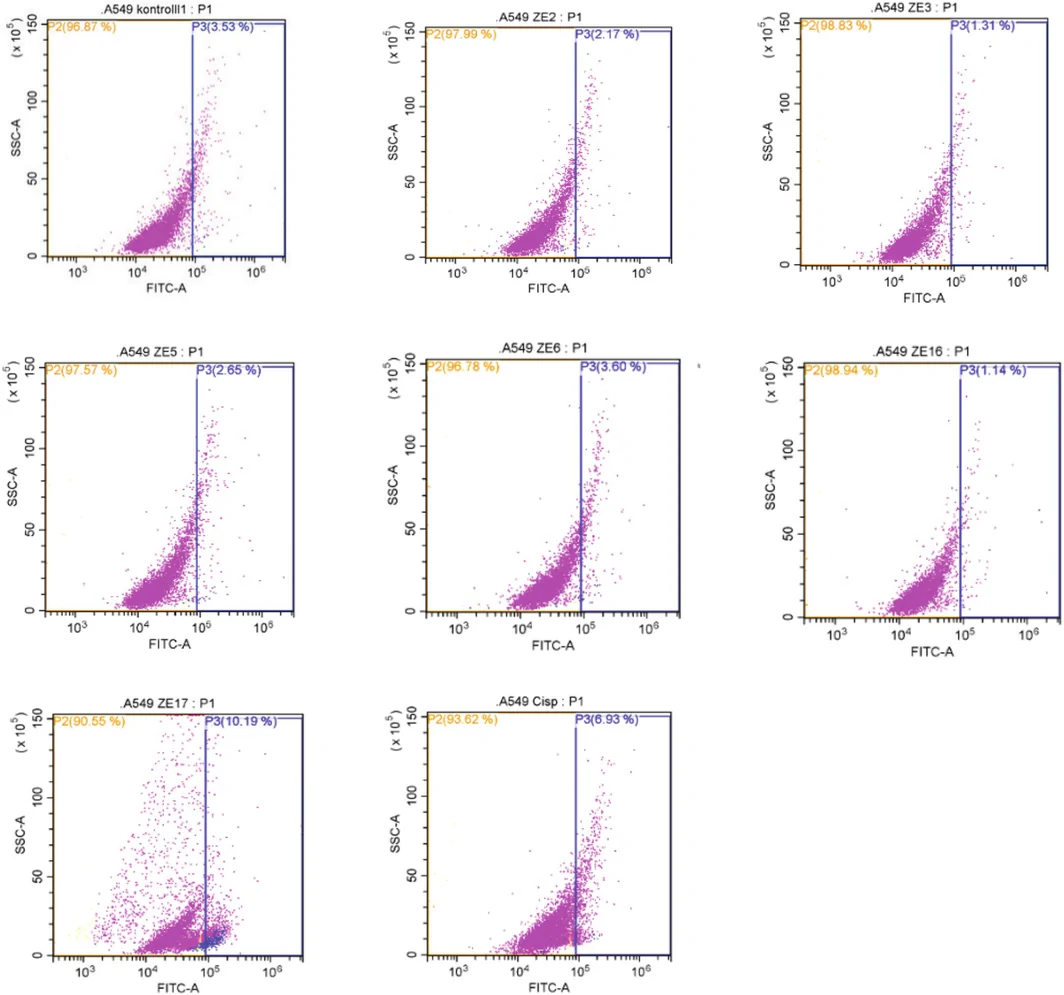
Caspase‐3 diagram for A549 cell line in **control 1**, **control 2**, and treated with **2b**, **2c**, **2e**, **2f**, **2k**, **2l** compounds and **cisplatin** respectively.

**FIGURE 4:**
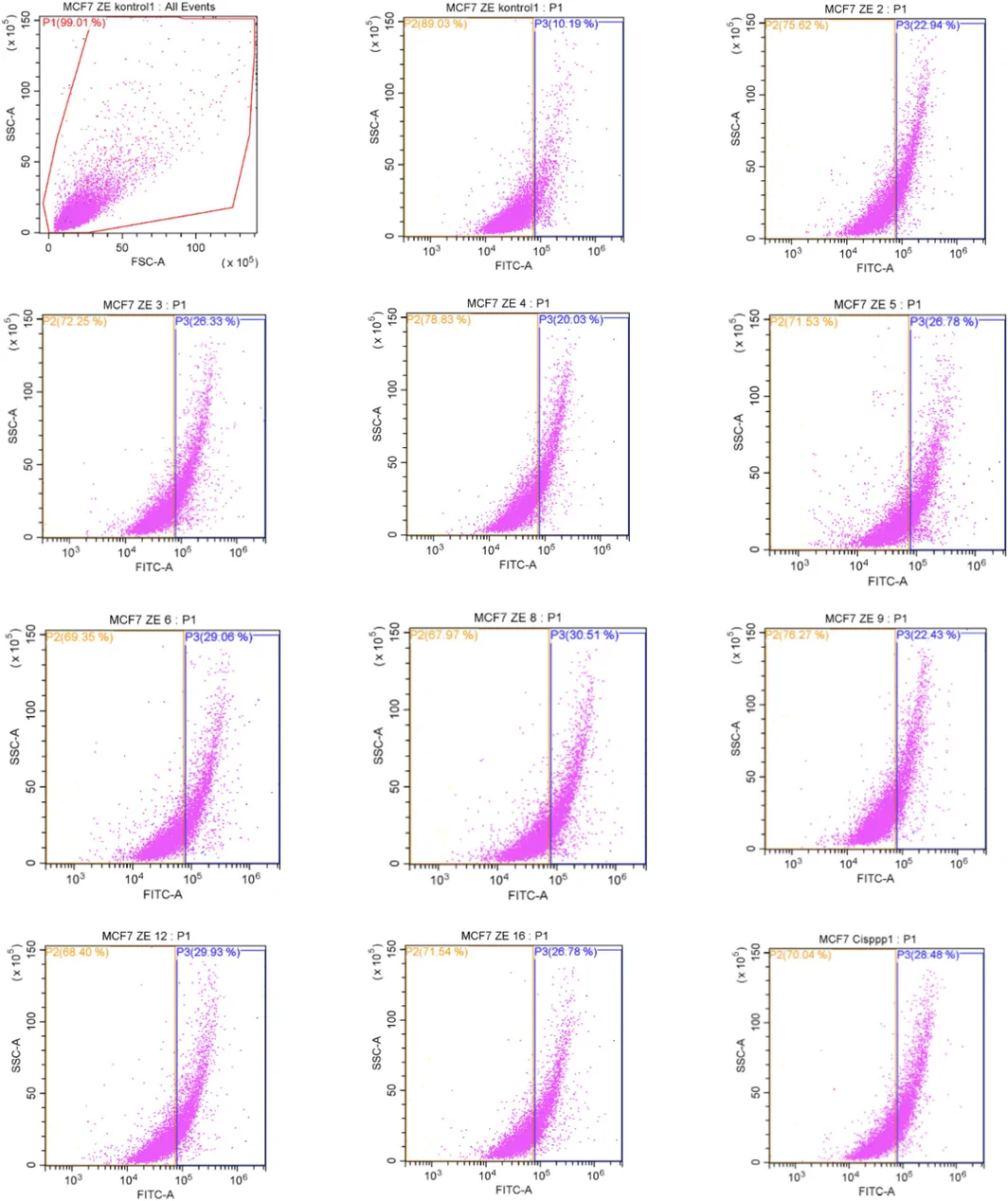
Caspase‐3 diagram for MCF‐7 cell line in **control 1**, **control 2**, and treated with **2b**, **2c**, **2d**, **2e**, **2f**, **2g**, **2h**, **2i**, **2k**, **2l** compounds and **cisplatin** respectively.

When the caspase‐3 activation levels on A549 cell line were examined, compound **2l** was recorded caspase‐3 activation ratio of 10.19%, representing a notable increase compared to cisplatin (6.93%). The caspase‐3 levels for tested compounds and the reference drug cisplatin on A549 cells are presented in Table 4 and Figure 3 on A549 cell line. In the analysis performed on MCF‐7 cell line, compounds **2f** (29.06%), **2g** (30.51%), **2i** (29.03%), and **2l** (30.20%) showed caspase‐3 activation levels that were slightly higher than those of cisplatin (28.48%). Additionally, compounds **2c**, **2e**, and **2k** also caused caspase‐3 activation with a percentage higher than 26.33% which is comparable to standard drug. The other compounds also provoked caspase‐3 activation higher than control group (**2b**, **2d**, and **2h**). The caspase‐3 activation percentages for tested compounds on MCF‐7 cells are summarized in Table 5 and Figure 4.

**TABLE 4:** Caspase‐3 activation levels (%) induced by synthesized compounds and cisplatin in A549 cells.

Compound	(+) Caspase‐3 activation P (%)	(−) Caspase‐3 activation P (%)
**2b**	2.17	97.99
**2c**	1.31	98.83
**2e**	2.65	97.57
**2f**	3.60	96.78
**2k**	1.14	98.94
**2l**	**10.19**	**90.55**
**Cisplatin**	**6.93**	**93.62**
**Control**	3.53	96.87

*Note:* Bold values show higher activity.

**TABLE 5:** Caspase‐3 activation levels (%) induced by synthesized compounds and cisplatin in MCF‐7 cells.

Compound	(+) Caspase‐3 activation P2 (%)	(−) Caspase‐3 activation P3 (%)
**2b**	22.94	75.62
**2c**	**26.33**	72.25
**2d**	20.03	78.83
**2e**	**26.78**	71.53
**2f**	**29.06**	69.35
**2g**	**30.51**	67.97
**2h**	22.43	76.27
**2i**	**29.93**	68.40
**2k**	**26.78**	71.54
**2l**	**30.20**	68.08
**Cisplatin**	**28.48**	70.04
**Control**	10.19	89.03

*Note:* Bold values show higher activity.

### 2.3 Computational Studies

#### 2.3.1 Molecular Docking

**FIGURE 5:**
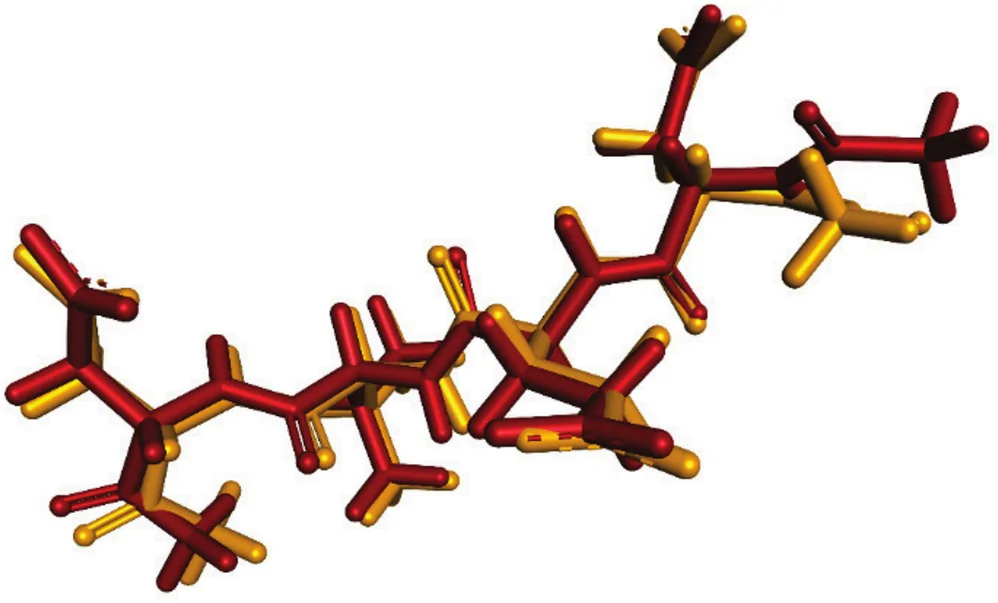
Superimposition of the crystallographic ligand pose (orange) and the redocked pose (red).

For the purpose of validating the molecular docking analysis, the ligand in the 4QTX crystal structure was subjected to a redocking procedure, and the RMSD between the crystallographic pose and the pose obtained from molecular docking was determined to be 0.8268 Å (Figure 5).

Molecular docking results showing the interactions of lowest binding energy poses obtained for each molecule are presented in Table 6.

**TABLE 6:** Interaction profiles of the lowest binding energy docking poses for each molecule.

Compound	Interactions
**2a**	Arg64 (H‐bond), Arg207 (salt bridge), His121 (H‐bond), Tyr204 (H‐bond), Phe250 (π‐π)
**2b**	Arg64 (H‐bond), Arg64 (salt bridge), Arg207 (salt bridge), Arg207 (π‐cation), His121 (H‐bond)
**2c**	Arg64 (H‐bond), Arg64 (salt bridge), Arg207 (salt bridge), Gln161 (H‐bond), His121 (H‐bond), Tyr204 (H‐bond)
**2d**	Arg64 (H‐bond), Arg64 (salt bridge), Arg207 (H‐bond), Arg207 (H‐bond), Arg207 (salt bridge), Phe250 (π‐π), Phe252 (π‐π)
**2e**	Arg64 (H‐bond), Arg64 (salt bridge), Arg207 (salt bridge), Gln161 (H‐bond), His121 (H‐bond), Phe250 (π‐π)
**2f**	Arg64 (H‐bond), Arg207 (salt bridge), Gln161 (H‐bond), His121 (H‐bond), Tyr204 (H‐bond)
**2g**	Arg64 (H‐bond), Arg64 (H‐bond), Arg207 (H‐bond), Arg207 (H‐bond), Gln161 (H‐bond), Tyr204 (π‐π), Phe250 (π‐π)
**2h**	Arg64 (H‐bond), Arg64 (salt bridge), Arg207 (salt bridge), Arg207 (salt bridge), Gln161 (H‐bond), His121 (H‐bond), Cys163 (H‐bond), Tyr204 (H‐bond), Phe256 (π‐π)
**2i**	Arg64 (H‐bond), Arg64 (salt bridge), Arg207 (salt bridge), His121 (H‐bond)
**2j**	Arg64 (H‐bond), Arg64 (salt bridge) Arg207 (salt bridge), Gln161 (H‐bond), His121 (H‐bond)
**2k**	Arg64 (H‐bond), Arg64 (salt bridge), Arg207 (salt bridge), His121 (H‐bond), Phe252 (π‐π)
**2l**	Arg64 (H‐bond), Arg64 (salt bridge), Arg207 (salt bridge), His121 (H‐bond), Trp206 (π‐π), Phe250 (π‐π)

**FIGURE 6:**
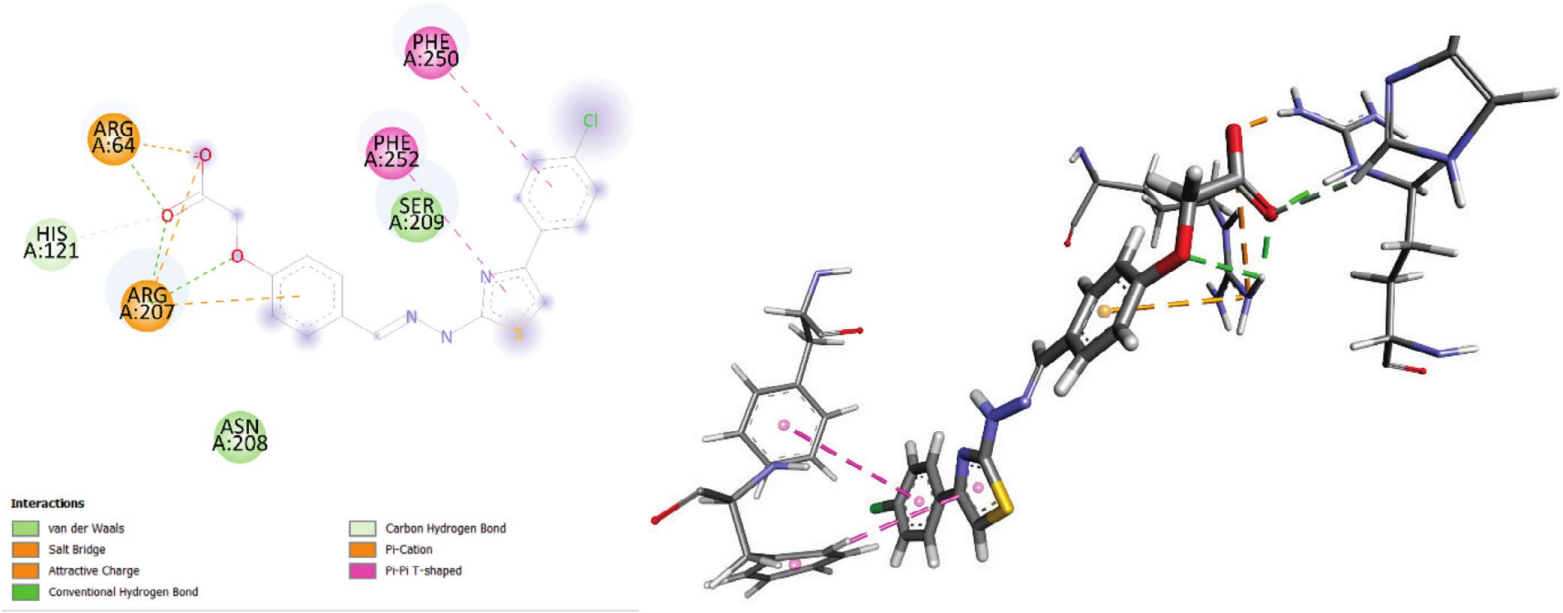
Two‐dimensional (2D) and three‐dimensional (3D) representations of the molecular docking analysis of compound **2d** with the 4QTX protein.

**FIGURE 7:**
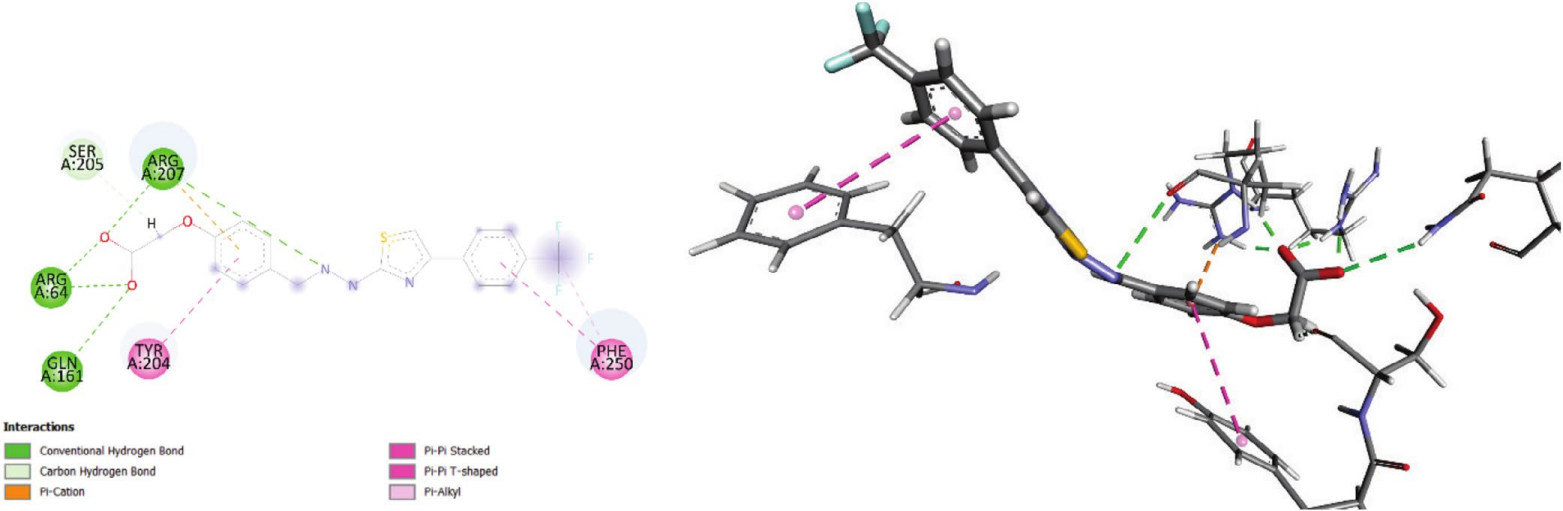
Two‐dimensional (2D) and three‐dimensional (3D) representations of the molecular docking analysis of compound **2g** with the 4QTX protein.

**FIGURE 8:**
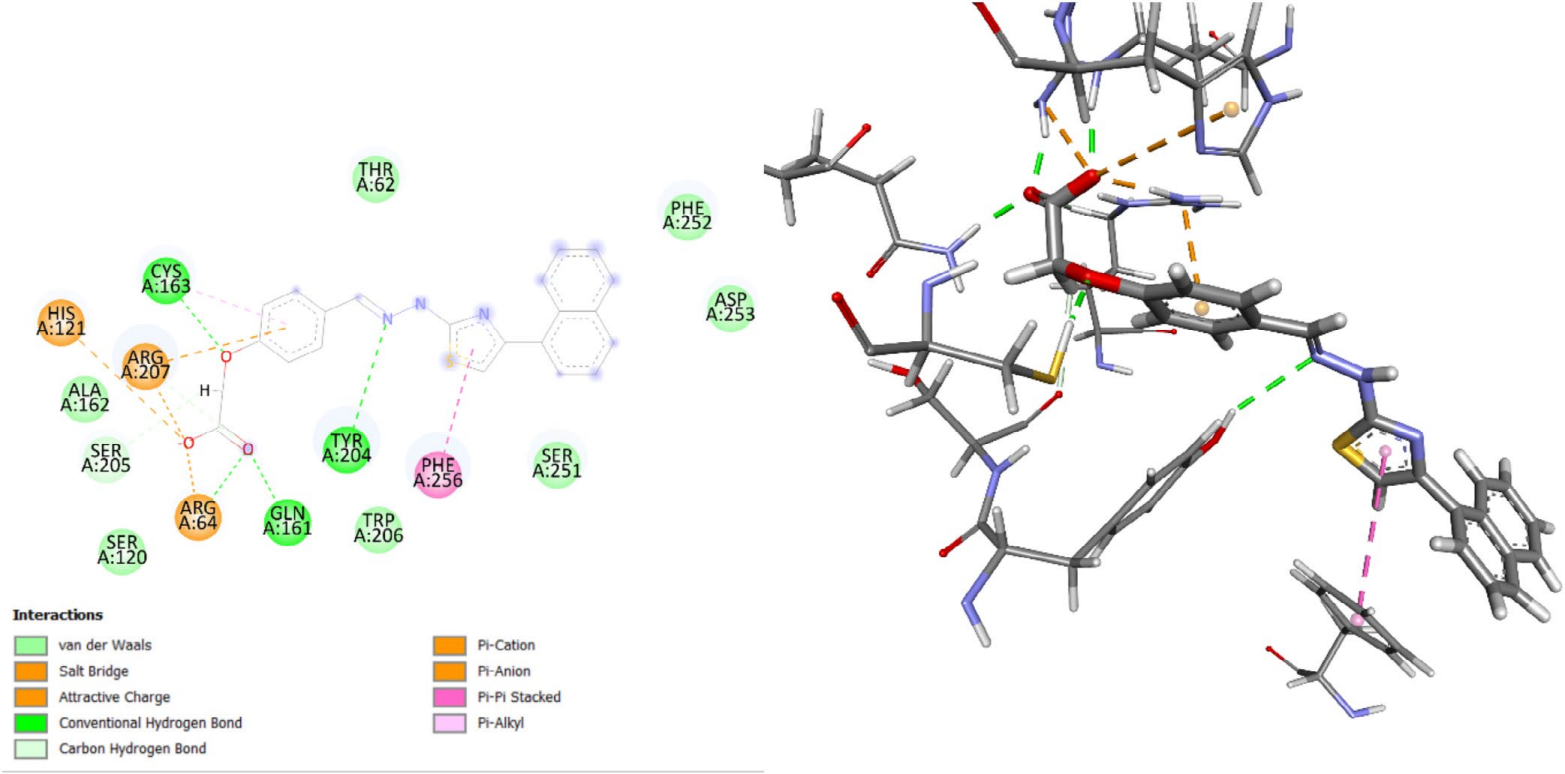
Two‐dimensional (2D) and three‐dimensional (3D) representations of the molecular docking analysis of compound **2l** with the 4QTX protein.

The molecular docking analysis showed that all compounds formed hydrogen‐bonding and salt bridge interactions with Arg64 and Arg207 through their carboxylate groups. In addition, most compounds exhibited hydrogen bonds with His121 and Gln161, as well as π–π interactions with Phe250 and Phe252. The 2D and 3D interaction patterns are shown for compound **2d** in Figure 6, for compound **2g** in Figure 7, and for compound **2l** in Figure 8.

#### 2.3.2 Molecular Dynamics Simulations

For the molecular dynamics simulations, the docked poses of compounds **2d**, **2g**, and **2l** together with the 4QTX protein structure were used as the initial complexes.

**FIGURE 9:**
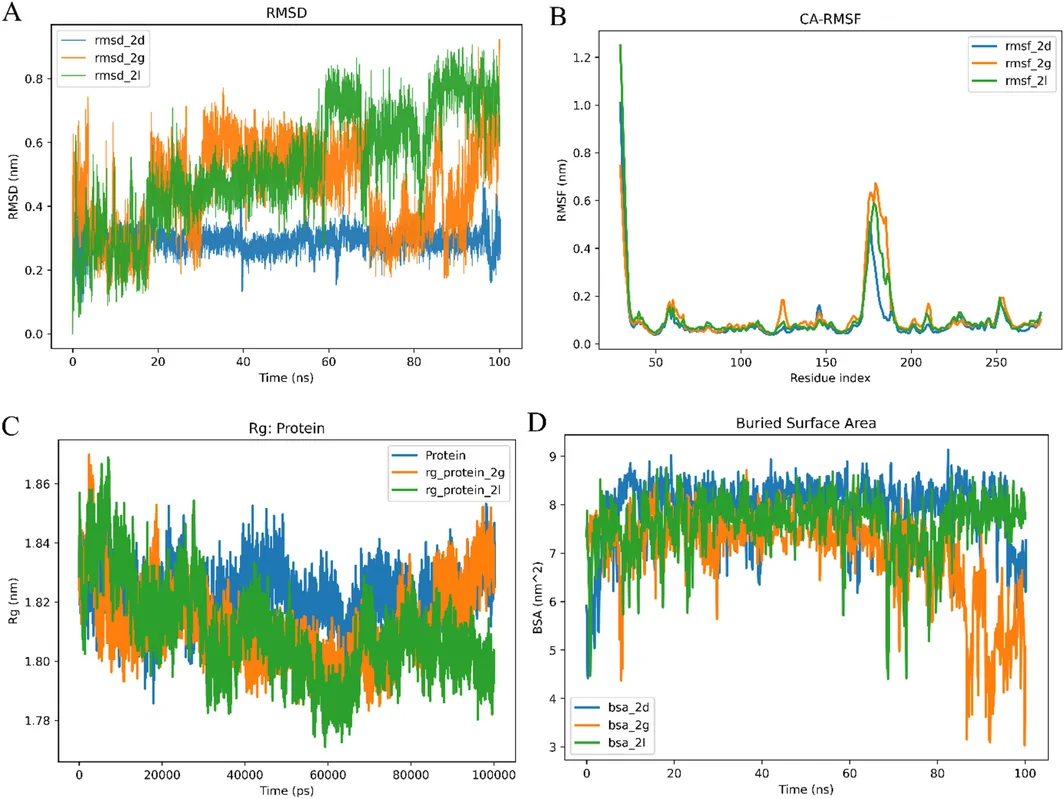
(A) RMSD profiles of the **2d**, **2g**, and **2l** compounds during the molecular dynamics simulation. (B) RMSF profiles of the protein backbone in complexes with **2d**, **2g**, and **2l**. (C) Rg profiles of the proteins in complexes with **2d**, **2g**, and **2l**. (D) Buried surface area (BSA) profiles of the **2d**, **2g**, and **2l** compounds in complex with the protein.

In Figure 9, analysis of the RMSD profiles of compounds **2d**, **2g**, and **2l** throughout the simulation demonstrated that **2d** was the most stable ligand, maintaining its binding pose over the entire simulation period. In contrast, although **2g** and **2l** remained within the active site, both compounds underwent transitions between alternative binding conformations. Comparison of the protein backbone RMSF profiles showed a slight increase in fluctuations within the 145–147 residue region in the presence of compound **2d**, whereas a similar increase was observed in the 121–125 residue region in the **2g**‐bound system. Notably, the 172–189 amino acid segment was most stable in the **2d**‐bound complex and least stable in the **2g**‐bound complex. Analysis of the radius of gyration (Rg) further indicated that the protein in complex with **2d** exhibited the lowest degree of fluctuation, while greater variations were observed in the **2g**‐ and **2l**‐bound systems. In terms of buried surface area (BSA), the **2g**‐bound complex displayed a pronounced decrease accompanied by marked fluctuations during the final 15 ns of the simulation, whereas the **2l**‐bound complex showed transient decreases between 60 and 80 ns. By comparison, only a slight reduction in BSA was observed during the final 10 ns of the **2d**‐bound system.

**FIGURE 10:**
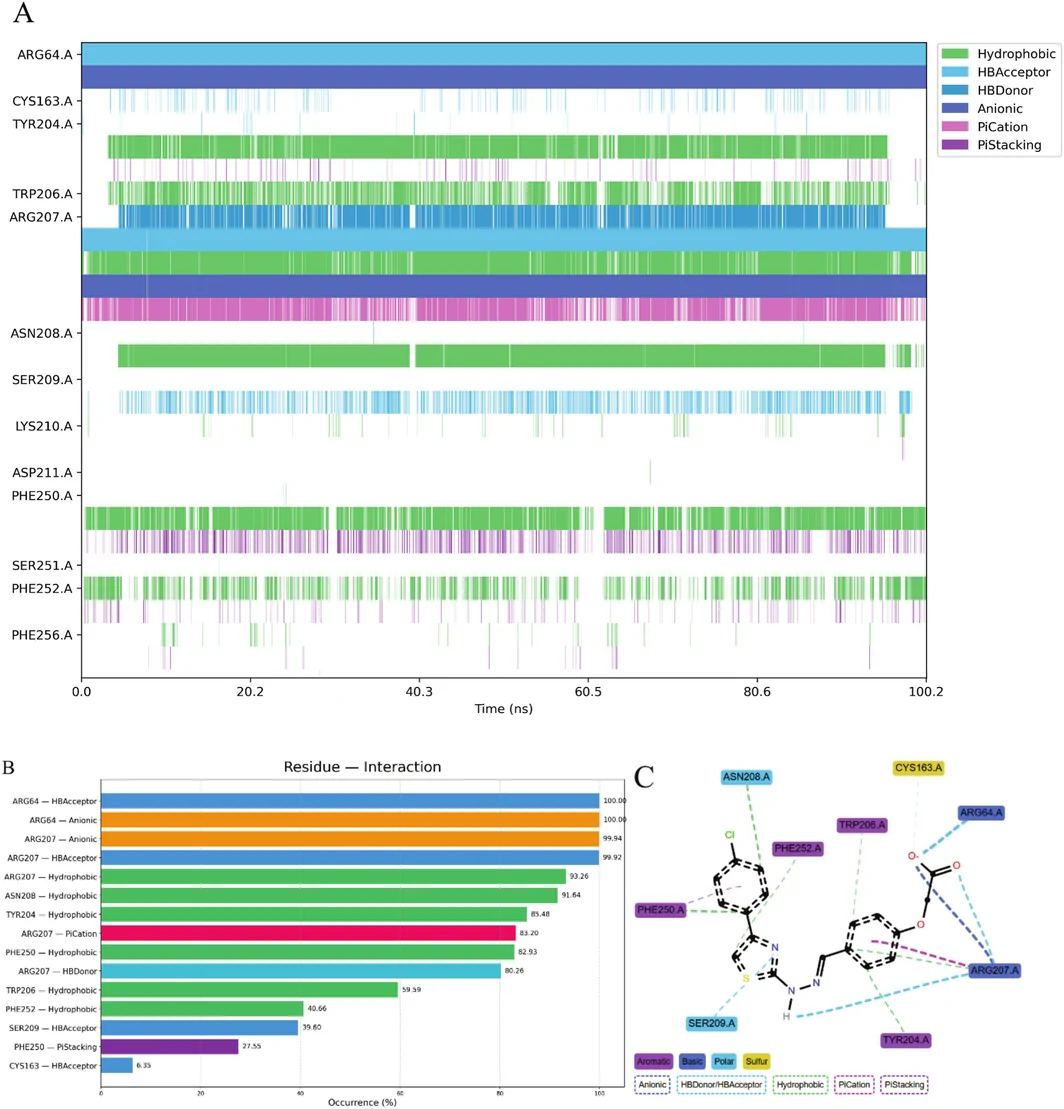
Protein–ligand interaction profile of the compound **2d** during the 100 ns molecular dynamics simulation. (A) Time‐resolved protein–ligand interaction map showing residue‐specific contacts throughout the simulation. (B) Occurrence frequencies of major residue–ligand interactions. (C) Two‐dimensional interaction diagram of the representative binding mode.

Figure 10 presents the time‐resolved interaction map, interaction frequencies, and two‐dimensional ligand–protein interaction diagram for compound **2d** throughout the simulation. The carboxylate group of compound **2d** formed stable two salt bridges and two hydrogen‐bond interactions with Arg64 and Arg207. Furthermore, the N–H group of the hydrazone moiety also participated in hydrogen‐bond formation. In addition, the phenyl ring engaged in a cation–π interaction with Arg207, whereas the thiazole group exhibited intermittent hydrogen‐bonding interactions with Ser209 during the simulation period.

**FIGURE 11:**
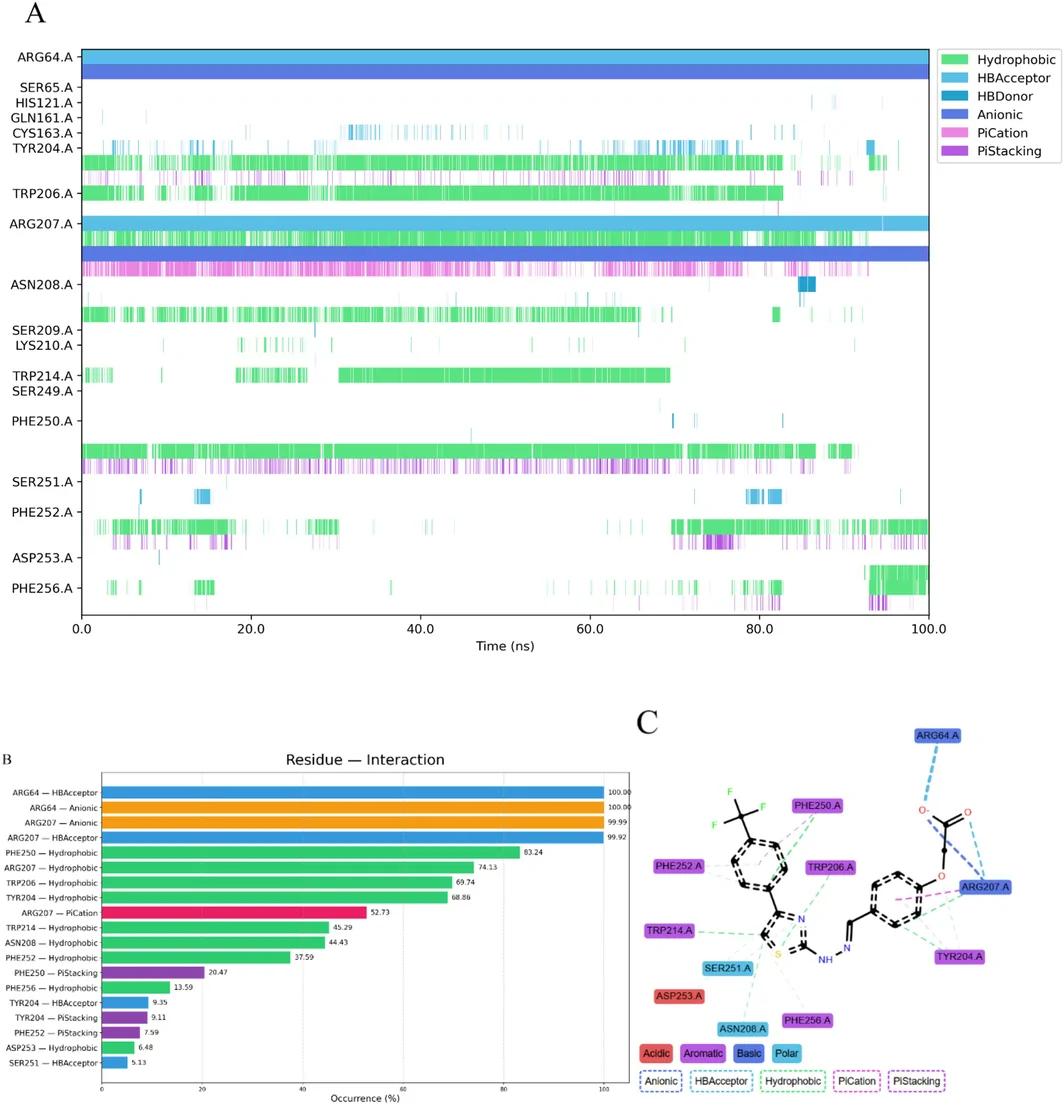
Protein–ligand interaction profile of the compound **2g** during the 100 ns molecular dynamics simulation. (A) Time‐resolved protein–ligand interaction map showing residue‐specific contacts throughout the simulation. (B) Occurrence frequencies of major residue–ligand interactions. (C) Two‐dimensional interaction diagram of the representative binding mode.

Figure 11 presents the time‐resolved interaction map, interaction frequencies, and two‐dimensional ligand–protein interaction diagram for compound **2g** throughout the simulation. Two salt bridges and two hydrogen bonds were observed between the carboxylate group of compound **2g** and the Arg64 and Arg207 residues. In addition, an intermittent cation–π interaction was formed between Arg207 and the phenyl ring. Intermittent π–π interactions were also detected with Tyr204, Phe250, and Phe252 at specific time intervals during the simulation. Tyr204 further exhibited occasional hydrogen‐bonding interactions. Notably, the hydrophobic interaction pattern of the ligand began to change after 60 ns, accompanied by a reduction in the overall number of interactions.

**FIGURE 12:**
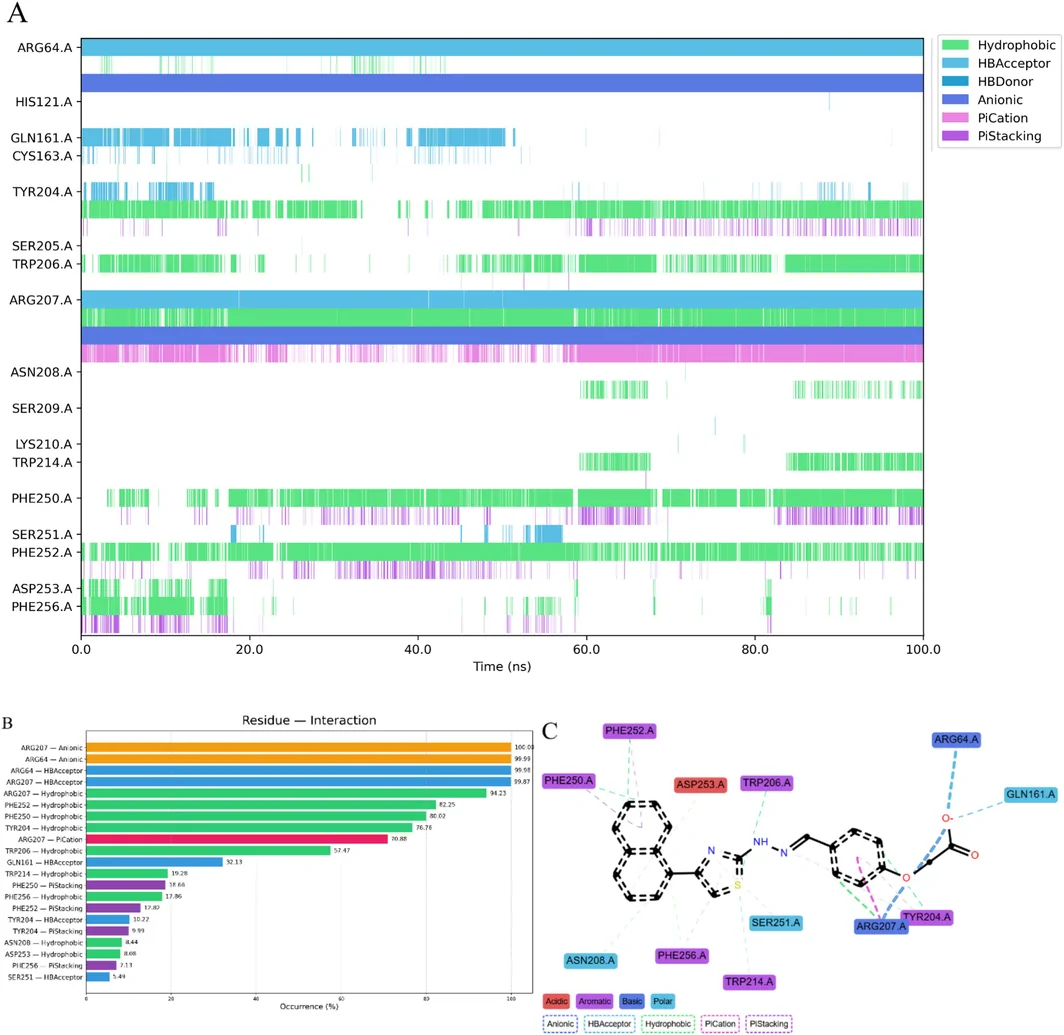
Protein–ligand interaction profile of the compound **2l** during the 100 ns molecular dynamics simulation. (A) Time‐resolved protein–ligand interaction map showing residue‐specific contacts throughout the simulation. (B) Occurrence frequencies of major residue–ligand interactions. (C) Two‐dimensional interaction diagram of the representative binding mode.

Figure 12 presents the time‐resolved interaction map, interaction frequencies, and two‐dimensional ligand–protein interaction diagram for compound **2l** throughout the simulation. Two salt bridges and two hydrogen bonds were observed between the carboxylate group of compound **2l** and the Arg64 and Arg207 residues. In addition, the phenyl ring formed a generally stable cation–pi interaction with Arg207, although this interaction became intermittent at certain time points. The carboxylate group also formed a hydrogen bond with Gln161 during approximately the first 50 ns of the simulation, after which this interaction disappeared. Intermittent π–π interactions with Phe250 and Phe252 were also detected at specific stages of the simulation. In the case of Tyr204, intermittent hydrogen‐bonding and π–π interactions were observed during the first 20 ns; however, these interactions gradually disappeared by 60 ns, after which only a more discontinuous π–π interaction persisted.

Analysis of the molecular dynamics simulation data revealed that the carboxylate groups of all three compounds established strong and stable interactions with Arg64 and Arg207. In addition, the common phenoxy moiety present in all structures displayed a pronounced cation–π interaction with Arg207, and these interactions remained stable throughout the simulation. In contrast, the thiazole ring and its attached aromatic substituents appeared to play a less prominent role, contributing mainly through hydrophobic contacts and intermittent π–π interactions during the simulation period. Furthermore, the hydrazone moiety formed hydrogen bonds in **2d**, hydrophobic contacts in **2l**, and no observable interaction in **2g**.

The persistent interactions of the common carboxylate group with Arg64 and Arg207 during the molecular dynamics simulations provide a plausible structural basis for the recognition of caspase‐3 by the synthesized compounds. Consistent with previous studies on structurally related thiazole derivatives, these findings suggest that interactions with residues involved in conformationally coupled loop and allosteric communication networks may contribute to caspase‐3 modulation.

The molecular dynamics simulation videos of the 4QTX protein–ligand complexes with compounds **2d**, **2g**, and **2l**, prepared using Visual Molecular Dynamics (VMD), are available at https://www.youtube.com/watch?v=g3bJaRdhCM8.

#### 2.3.3 Density Functional Theory (DFT) Analysis

**FIGURE 13:**
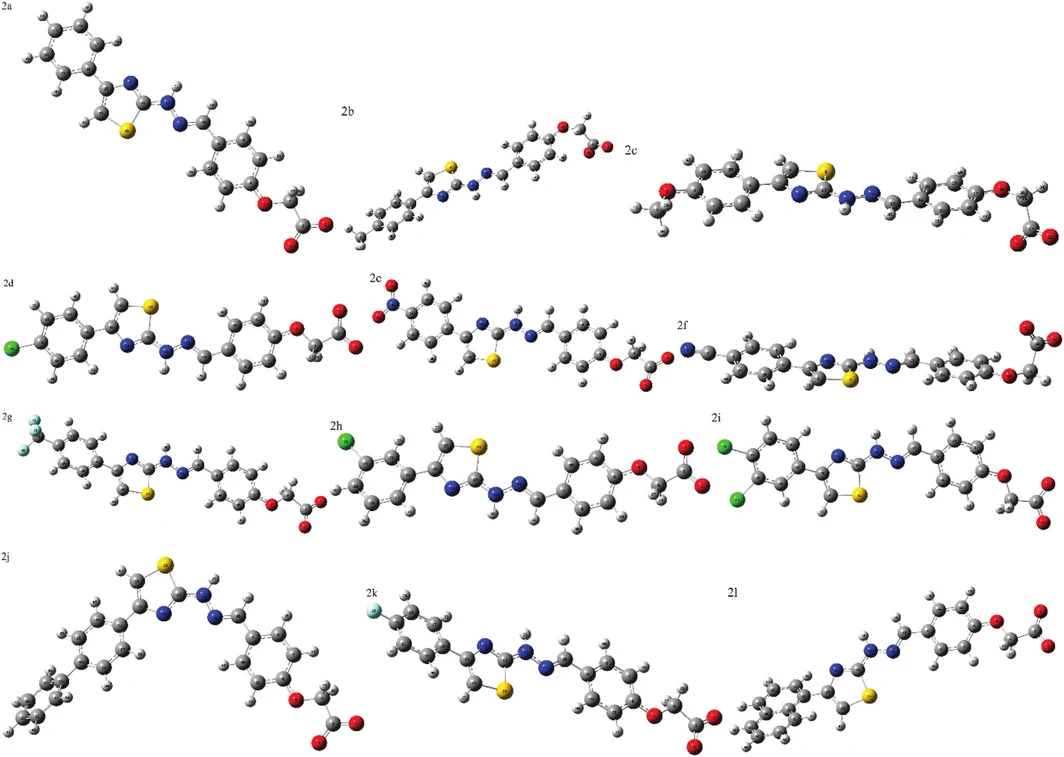
Optimized geometries of the carboxylate forms of all final compounds obtained at the B3LYP/6‐31+G(d,p) level in water using the SMD solvation model.

DFT optimization afforded stable minimum‐energy geometries for the carboxylate forms of all final compounds, and the corresponding optimized structures are presented in Figure 13.

The polarizability, dipole moment, electronic energy, E_HOMO_, E_LUMO_, and E_LUMO_ − E_HOMO_ values obtained from the DFT analyses for all final compounds are presented in Table 7.

**TABLE 7:** DFT descriptors (polarizability, dipole moment, electronic energy, E_HOMO_, E_LUMO_, and E_LUMO_ − E_HOMO_) for the final compounds.

Compound	Polarizability (a.u.)	Dipole moment (Debye)	Electronic energy (Hartree)	E_HOMO_ (Hartree)	E_LUMO_ (Hartree)	E_LUMO_ − E_HOMO_ (Hartree)
**2a**	451.197	49.833	**−1482.682**	−0.19628	−0.05746	0.13882
**2b**	471.851	60.719	−1522.002	−0.19488	−0.05687	0.13801
**2c**	479.369	58.408	−1597.213	−0.19334	−0.05659	0.13675
**2d**	468.520	**23.188**	−1942.277	−0.19689	−0.05840	0.13849
**2e**	533.444	59.900	−1687.197	−0.20070	**−0.11554**	**0.08516**
**2f**	490.484	56.076	−1574.932	−0.19875	**−0.07092**	**0.12783**
**2g**	470.435	51.916	−1819.740	−0.19848	−0.06101	0.13747
**2h**	467.259	**66.545**	−1942.276	−0.19776	−0.05866	**0.13910**
**2i**	483.907	**24.214**	**−2401.866**	−0.19817	−0.06063	0.13754
**2j**	**574.022**	57.619	−1713.750	−0.19553	−0.05623	**0.13930**
**2k**	**447.504**	**21.239**	−1581.922	−0.19644	−0.05750	0.13894
**2l**	531.463	57.052	−1636.328	−0.19596	−0.05827	0.13769

*Note:* Bold values show higher activity.

The dipole moment values ranged from 21.239 to 66.545 D, suggesting that the compounds are highly polar as a result of their ionic character. Among them, compound **2h**, bearing a 3‐chloro substituent, showed the highest dipole moment, whereas compounds with 4‐chloro, 3,4‐dichloro, and 4‐phenyl substituents (**2d**, **2i**, and **2k**) exhibited comparatively lower values. In terms of polarizability, compound **2j** had the highest value, which may be attributed to its more extended conjugated π‐electron system, while the 4‐fluoro derivative exhibited the lowest polarizability. In terms of electronic energy, compound **2i** was found to possess the lowest value by a clear margin, whereas compound **2a** exhibited the highest electronic energy. Evaluation of the HOMO energy levels suggested that compound **2e** would be markedly less prone to oxidation than the other compounds, while at the same time being more susceptible to reduction. Compound **2f** also displayed a greater tendency toward reduction, albeit less pronounced than that of **2e**, whereas its oxidation tendency remained similar to that of the other compounds. Analysis of the HOMO–LUMO energy gaps demonstrated that compound **2e**, containing a nitro substituent, was distinctly more easily excitable than the other compounds. Similarly, compound **2f**, bearing a cyano group, also showed a greater tendency toward excitation relative to the rest of the series. With the exception of **2e** and **2f**, all compounds displayed comparable HOMO–LUMO energy profiles. The frontier molecular orbitals were predominantly distributed over the thiazole and phenyl groups linked to the hydrazone moiety, together with the phenoxy oxygen, in all structures except **2e**, **2f**, and **2g**. In compound **2e**, the LUMO was primarily localized on the substituent‐bearing phenyl ring and, to a lesser extent, on the thiazole ring. In compound **2f**, the LUMO was distributed over the same phenyl ring and the thiazole moiety, with a minor extension onto the hydrazone group. By contrast, in compound **2g**, the LUMO exhibited a broader distribution throughout the molecule, although it remained predominantly centered on the substituted phenyl ring. The carboxylate group did not contribute significantly to the HOMO or LUMO in any of the structures. The HOMO and LUMO isosurface plots of all compounds are provided in Supporting Information S2.

Assessment of the Hirshfeld charge and condensed Fukui function analyses revealed that the carboxylate group did not contribute significantly to the overall reactivity of the compounds. In all structures except **2e** and **2f**, the hydrazone carbon atom was identified as the site with the highest electrophilic character (f^+^). In compound **2e**, electrophilic character was localized on the NO_2_ group, whereas in compound **2f** it was distributed over the cyano substituent and the phenyl ring to which it is attached. For all compounds, nucleophilic character was localized at the C5 atom of the thiazole ring and at the nitrogen atom attached to the 2‐position of the thiazole ring. An increase in the electron‐withdrawing nature of the R substituent reduced the nucleophilicity of the thiazole C5 atom, whereas a stronger electron‐donating character enhanced it. The Fukui f^+^ values of the hydrazone C, along with the f^−^ values of thiazole C5 and hydrazone NH, are listed in Table 8. The Hirshfeld charges and condensed Fukui function values (f^+^, f^−^, and f^0^) for all compounds are provided in Supporting Information S2.

**TABLE 8:** Fukui indices of selected corresponding atoms presents in final compounds: f^+^ at the hydrazone carbon, and f^−^ at thiazole C5 and the hydrazone NH.

Molecule	f^+^ (hydrazone C)	f^−^ (hydrazone NH)	f^−^ (thiazole's C5)
**2a**	0.201725	0.158343	0.143779
**2b**	0.201492	0.152221	0.150912
**2c**	0.201603	0.139544	0.161526
**2d**	0.202050	0.160006	0.139071
**2e**	0.003506	0.166535	0.118088
**2f**	0.008565	0.165460	0.124874
**2g**	0.107858	0.165323	0.129960
**2h**	0.202065	0.163766	0.134265
**2i**	0.199263	0.164397	0.130022
**2j**	0.203619	0.147470	0.141739
**2k**	0.202089	0.158225	0.144208
**2l**	0.198933	0.163352	0.132420

*Note:* Hirshfeld atomic charges were calculated with hydrogen charges summed into the corresponding heavy atoms.

## 3 Conclusion

In this study, 12 novel phenoxyacetic acid‐containing 4‐arylthiazole–hydrazone derivatives (**2a–l**) were synthesized from 4‐formylphenoxyacetic acid and evaluated for their antiproliferative effects against MCF‐7 breast cancer, A549 lung cancer, and L929 non‐malignant fibroblast cells. The compound series exhibited a marked cell‐line‐dependent activity profile, with several derivatives showing greater antiproliferative activity against MCF‐7 cells than cisplatin while retaining lower cytotoxicity toward L929 cells. In particular, compounds **2d** and **2i** emerged as promising MCF‐7‐selective lead structures, although their potency and selectivity require confirmation using additional concentration points and independent biological experiments.

A preliminary structure–activity relationship was evident from the antiproliferative findings. In MCF‐7 cells, para‐chloro substitution in compound **2d** and 3,4‐dichloro substitution in compound **2i** were associated with the most favorable activity profiles. The low‐micromolar activities of the biphenyl derivative **2j**, the 4‐fluoro derivative **2k**, and the naphthyl derivative **2l** further suggest that selected halogenated, hydrophobic, and extended aromatic substituents may favor activity against this cell line. By contrast, the 4‐methoxy derivative **2c**, the 4‐nitro derivative **2e**, and the 4‐cyano derivative **2f** displayed the most favorable activities in A549 cells. These findings indicate that the identity, position, size, and physicochemical properties of the aryl substituent attached to the thiazole ring are important determinants of cell‐line preference. However, because the present series contains a limited number of structurally related derivatives, these observations should be regarded as a preliminary SAR that requires further analogue development.

Regarding the Annexin V results in MCF‐7 cells, compounds **2d**, **2c**, and **2h** induced total apoptotic cell populations of approximately 24%–25%, whereas compound **2i** produced a lower apoptotic response of 15.68%. Although these values remained below that of cisplatin (31.58%), they indicate that several derivatives promoted apoptosis‐associated cell death. Intracellular active‐caspase‐3 staining further showed positive cell populations of approximately 22%–31% for the tested compounds. Compounds **2f**, **2g**, and **2i** produced active‐caspase‐3‐positive percentages comparable to or slightly higher than that observed with cisplatin, whereas compounds **2c**, **2d**, and **2h** also showed increases relative to the untreated control.

The final compounds were evaluated on caspase‐3 protein with the molecular docking analysis and it has been observed that hydrogen bonding and salt bridges are formed with Arg64 and Arg207 via carboxylate groups in all compounds. Molecular dynamics simulation data supported that the carboxylate groups of studied three compounds (**2d**, **2g**, and **2l**) formed strong and stable interactions with Arg64 and Arg207. The conserved interactions involving Arg64 and Arg207, together with their persistence during the molecular dynamics simulations, support the structural plausibility of caspase‐3 recognition by the synthesized compounds. This interpretation is consistent with previous studies reporting that structurally related thiazole derivatives may interact with residues involved in caspase‐3 loop and allosteric communication networks (Evren et al. 2024; Kaya et al. 2025). DFT analyses revealed that the aryl substituents influenced the electronic properties and local reactivity of the compounds, particularly their dipole moments, polarizabilities, frontier molecular orbital energies, and Fukui indices.

The novelty of this study lies not only in the synthesis of a new phenoxyacetic acid‐containing 4‐arylthiazole–hydrazone series but also in the identification of markedly different structure–activity profiles in breast and lung cancer cells. The observed cell‐line‐dependent structure–activity relationship demonstrates that modification of the aryl substituent can influence antiproliferative activity and selectivity within this compound series. These findings provide a rational basis for the design of more selective analogues and support the consideration of these compounds as preliminary lead structures for further anticancer drug‐development studies.

## 4 Experimental

### 4.1 Chemistry

All chemicals were purchased from Merck Chemicals (Merck KGaA, Darmstadt, Germany), Fluka (Germany) and Sigma‐Aldrich Corp. St. Louis, MO, USA. Reaction completion was followed by thin layer chromatography (TLC) on silica gel 60 F_254_ aluminum plates. MP90 digital melting point device (Mettler Toledo, Switzerland) was used to determine the melting points of the synthesized molecules. For ^1^H NMR, ^13^C NMR, and IR analyses, Bruker, UltraShield 300 MHz (USA), and Shimadzu‐IR Affinity‐IS (Japan) instruments were used, respectively, and recorded in DMSO‐*d*_*6*_. Finally, for mass spectrometry analysis, results were recorded using the Advion Compact Mass Spectrometer (USA).

### 4.2 Synthesis of the Compounds

#### 4.2.1 2‐[4‐((2‐Carbamothioylhydrazono)methyl)phenoxy]acetic acid (**1**)

Equal molar amounts of thiosemicarbazide (0.0141 mol, 1.29 g) and 4‐formylphenoxyacetic acid (0.0141 mol, 2.55 g) were added and refluxed in 50 mL ethanol for 5 h. The reaction was monitored by thin‐layer chromatography. After the reaction was complete, the raw material, which settled when it cooled, was filtered, then washed with ethanol. The precipitate was dried to obtain the crude product.

#### 4.2.2 Synthesis of 2‐[4‐[(2‐(4‐substituted thiazol‐2‐yl)hydrazono)methyl]phenoxy]acetic acid Derivatives (**2a–l**)

In the second step, the 2‐[4‐((2‐carbamothioylhydrazono)methyl)phenoxy]acetic acid (0.0012 mol, 0.3 g) (**1**) obtained in the first step was weighed in equivalent proportions to the phenacyl bromide derivatives and refluxed with various equivalent moles of aryl aldehyde derivatives in ethanol for 3 h. At the end of the 3 h reaction, the compound was checked using thin‐layer chromatography (TLC); the settled portion was filtered and washed with ethanol.

##### 4.2.2.1 2‐[4‐[(2‐(4‐Phenylthiazol‐2‐yl)hydrazono)methyl]phenoxy]acetic acid (**2a**)

Yield: 71%. M.P.: 212°C–218°C. IR ν (cm^−1^): 2965, 2884 (Aliphatic C‐H), 1767 (C=O), 1620–1409 (C=C, C=N), 1302–1070 (C‐O, C‐N), 881–704 (benzene ring out‐of‐plane deformation band). ^1^H‐NMR (300 MHz, DMSO‐*d*_*6*_), δ: 4.74 (s, 2H, CH_2_), 6.99 (d, 2H, J: 7.77 Hz, phenoxy C2,6‐H), 7.31 (brs, 2H, thiazole C5‐H and phenyl C4‐H), 7.41 (t, 2H, J: 7.38 Hz, phenyl C3,5‐H), 7.60 (d, 2H, J: 7.77 Hz, phenoxy C3,5‐H), 7.85 (d, 2H, J: 7.53 Hz, phenyl C2,6‐H), 8.00 (s, 1H, CH=N), 12.10 (NH). ^13^C‐NMR (75 MHz, DMSO‐*d*_*6*_), δ: 64.97, 103.89, 115.35, 125.99, 127.95, 128.01, 128.21, 129.07, 135.06, 141.75, 150.73, 159.19, 168.79, 170.44. APCI (m/z): [M+H]^+^: Theoretical value for C_18_H_15_N_3_O_3_S: 354.39, experimental value: 354.40.

##### 4.2.2.2 2‐[4‐[(2‐(4‐(4‐Methylphenyl)thiazol‐2‐yl)hydrazono)methyl]phenoxy]acetic acid (**2b**)

Yield: 68%. M.P.: 222°C–227°C. IR ν (cm^−1^): 3113, 3070 (Aromatic C‐H), 2914 (Aliphatic C‐H), 1643 (C=O), 1606–1402 (C=C, C=N), 1252–1061 (C‐O, C‐N), 822–736 (benzene ring out‐of‐plane deformation band). ^1^H‐NMR (300 MHz, DMSO‐*d*_*6*_), δ: 2.31 (s, 3H, CH_3_), 4.73 (s, 2H, CH_2_), 6.97 (d, 2H, J: 7.77 Hz, phenoxy C2,6‐H), 7.19–7.21 (m, 3H, thiazole C5‐H and phenyl C3,5‐H), 7.58 (d, 2H, J: 7.77 Hz, phenyl C2,6‐H), 7.74 (d, 2H, J: 7.53 Hz, phenoxy C3,5‐H), 7.97 (s, 1H, CH=N), 12.42 (NH). ^13^C‐NMR (75 MHz, DMSO‐*d*_*6*_), δ: 21.27 (CH_3_), 64.97 (CH_2_), 102.92, 115.31, 125.92, 127.71, 129.29, 132.55, 137.21, 141.49, 150.99, 159.14, 159.60, 168.69, 170.45. APCI (m/z): [M+H]^+^: Theoretical value for C_19_H_17_N_3_O_3_S: 368.42, experimental value: 368.4.

##### 4.2.2.3 2‐[4‐[(2‐(4‐(4‐Methoxyphenyl)thiazol‐2‐yl)hydrazone)methyl]phenoxy]acetic acid (**2c**)

Yield: 61%. M.P.: 252°C–258°C. IR ν (cm^−1^): 3115, 3003 (Aromatic C‐H), 2938, 2839 (Aliphatic C‐H), 1634 (C=O), 1608–1404 (C=C, C=N), 1298–1063 (C‐O, C‐N), 947–709 (benzene ring out‐of‐plane deformation band). ^1^H‐NMR (300 MHz, DMSO‐*d*_*6*_), δ: 3.79 (s, 3H, OCH_3_), 4.74 (s, 2H, CH_2_), 6.96–6.99 (m, 4H, J: 7.77 Hz, phenoxy C2,6‐H and phenyl C3,5‐H), 7.13 (s, 1H, thiazole C5‐H), 7.60 (d, 2H, J: 7.51 Hz, phenyl C2,6‐H), 7.78 (d, 2H, J: 7.51 Hz, phenoxy C3,5‐H), 7.98 (s, 1H, CH=N), 12.17 (NH). ^13^C‐NMR (75 MHz, DMSO‐*d*_*6*_), δ: 55.58 (CH_3_), 64.97 (CH_2_), 101.71, 114.42, 115.34, 127.30, 128.02, 128.05, 128.15, 141.48, 150.71, 159.14, 159.23, 168.67, 170.45. APCI (m/z): [M+H]^+^: Theoretical value for C_19_H_17_N_3_O_4_S: 384.42, experimental value: 384.3.

##### 4.2.2.4 2‐[4‐[(2‐(4‐(4‐Chlorophenyl)thiazol‐2‐yl)hydrazono)methyl]phenoxy]acetic acid (**2d**)

Yield: 72%. M.P.: 236°C–255°C (decomposed). IR ν (cm^−1^): 3115 (Aromatic C‐H), 2987, 2899 (Aliphatic C‐H), 1608–1404 (C=C, C=N), 1225–1070 (C‐O, C‐N), 916–704 (benzene ring out‐of‐plane deformation band). ^1^H‐NMR (300 MHz, DMSO‐*d*_*6*_), δ: 4.74 (s, 2H, CH_2_), 6.99 (d, 2H, J: 7.90 Hz, phenoxy C2,6‐H), 7.38 (s, 1H, thiazole C5‐H), 7.46 (d, 2H, J: 7.32 Hz, phenyl C3,5‐H), 7.59 (d, 2H, J: 7.65 Hz, phenoxy C3,5‐H), 7.87 (d, 2H, J: 7.65 Hz, phenyl C2,6‐H), 7.99 (s, 1H, CH=N), 12.06 (NH). ^13^C‐NMR (75 MHz, DMSO‐*d*_*6*_), δ: 64.97 (CH_2_), 104.69, 115.35, 127.68, 127.92, 128.21, 129.08, 132.34, 134.06, 141.76, 149.72, 159.20, 168.92, 170.44. APCI (m/z): [M+H]^+^: Theoretical value for C_18_H_14_ClN_3_O_3_S: 388.84, experimental value: 388.5.

##### 4.2.2.5 2‐[4‐[(2‐(4‐(4‐Nitrophenyl)thiazol‐2‐yl)hydrazono)methyl]phenoxy]acetic acid (**2e**)

Yield: 68%. M.P.: 259°C–265°C. IR *ν* (cm^−1^): 3126, 3001 (Aromatic C‐H), 2910 (Aliphatic C‐H), 1599–1344 (C=C, C=N), 1242–1051 (C‐O, C‐N), 850–708 (benzene ring out‐of‐plane deformation band). ^1^H‐NMR (300 MHz, DMSO‐*d*_*6*_), δ: 4.74 (s, 2H, CH_2_), 6.99 (d, 2H, J: 8.09 Hz, phenoxy C2,6‐H), 7.61 (d, 2H, J: 8.09 Hz, phenoxy C3,5‐H), 7.71 (s, 1H, thiazole C5‐H), 8.01 (s, 1H, CH=N), 8.11 (d, 2H, J: 8.63 Hz, phenyl C2,6‐H), 8.28 (d, 2H, J: 8.17 Hz, phenyl C3,5‐H), 12.17 (NH). ^13^C‐NMR (75 MHz, DMSO‐*d*_*6*_), δ: 64.97 (CH_2_), 108.81, 115.36, 124.58, 126.79, 127.82, 128.29, 141.18, 142.16, 146.65, 148.97, 159.28, 169.21, 170.43. APCI (m/z): [M+H]^+^: Theoretical value for C_18_H_14_N_4_O_5_S: 399.39, experimental value: 399.3.

##### 4.2.2.6 2‐[4‐[(2‐(4‐(4‐Cyanophenyl)thiazol‐2‐yl)hydrazono)methyl]phenoxy]acetic acid (**2f**)

Yield: 59%. M.P.: 223°C–224°C. IR *ν* (cm^−1^): 3103 (Aromatic C‐H), 2986, 2880 (Aliphatic C‐H), 1714 (C=O), 1605–1404 (C=C, C=N), 1258–1060 (C‐O, C‐N), 833–737 (benzene ring out‐of‐plane deformation band) ^1^H‐NMR (300 MHz, DMSO‐*d*_*6*_), δ: 4.74 (s, 2H, CH_2_), 6.99 (d, 2H, J: 7.74 Hz, phenoxy C2,6‐H), 7.59–7.62 (m, 3H, thiazole C5‐H and phenyl C3,4‐H), 7.86 (d, 2H, J: 7.98 Hz, phenoxy C3,5‐H), 8.01–8.04 (m, 3H, phenyl C2,6‐H and CH=N), 12.13 (NH). ^13^C‐NMR (75 MHz, DMSO‐*d*_*6*_), δ: 64.97 (CH_2_), 107.78, 110.01, 115.13, 115.35, 119.47, 126.57, 127.84, 129.29, 133.15, 139.26, 142.06, 149.25, 159.25, 169.11, 170.43. APCI (m/z): [M+H]^+^: Theoretical value for C_19_H_14_N_4_O_3_S: 379.40, experimental value: 379.3.

##### 4.2.2.7 2‐[4‐[(2‐(4‐(4‐Trifluoromethylphenyl)thiazol‐2‐yl)hydrazono)methyl]phenoxy]acetic acid (**2g**)

Yield: 71%. M.P.: 255°C–257°C. IR *ν* (cm^−1^): 3647 (O‐H), 2972 (Aliphatic C‐H), 1506–1406 (C=C, C=N), 1312–1057 (C‐O, C‐N), 829–723 (benzene ring out‐of‐plane deformation band). ^1^H‐NMR (300 MHz, DMSO‐*d*_*6*_), δ: 4.74 (s, 2H, CH_2_), 6.99 (d, 2H, J: 8.08 Hz, phenoxy C2,6‐H), 7.55 (s, 1H, thiazole C5‐H), 7.60 (d, 2H, J: 8.47 Hz, phenyl C3,5‐H), 7.76 (d, 2H, J: 8.47 Hz, phenoxy C3,5‐H), 8.00 (s, 1H, CH=N), 8.06 (d, 2H, J: 7.76 Hz, phenyl C2,6‐H), 12.14 (NH). ^13^C‐NMR (75 MHz, DMSO‐*d*_*6*_), δ: 64.97 (CH_2_), 106.68, 115.35, 123.48, 126.06, 126.18, 127.82, 128.24, 138.87, 141.93, 149.44, 159.23, 169.08, 170.44. APCI (m/z): [M+H]^+^: Theoretical value for C_18_H_15_N_3_O_3_S: 354.39, experimental value: 354.4.

##### 4.2.2.8 2‐[4‐[(2‐(4‐(3‐Chlorophenyl)thiazol‐2‐yl))hydrazono)methyl]phenoxy]acetic acid (**2h**)

Yield: 70%. M.P.: 205°C–209°C. IR *ν* (cm^−1^): 3109, 3070 (Aromatic C‐H), 2988, 2900, 2872 (Aliphatic C‐H), 1732 (C=O), 1630–1379 (C=C, C=N), 1209–1074 (C‐O, C‐N), 880–750 (benzene ring out‐of‐plane deformation band). ^1^H‐NMR (300 MHz, DMSO‐*d*_*6*_), δ: 4.74 (s, 2H, CH_2_), 6.97 (brs, 2H, phenoxy C2,6‐H), 7.34–7.35 (m, 1H, phenyl C3‐H), 7.41–7.44 (m, 2H, thiazole C5‐H and phenyl C4‐H), 7.58 (d, 2H, J: 6.52 Hz, phenoxy C3,5‐H), 7.82 (d, 1H, J: 7.31 Hz, phenyl C2,6‐H), 7.90 (brs, 1H, phenyl C2‐H), 8.00 (s, 1H, CH=N), 12.03 (NH). ^13^C‐NMR (75 MHz, DMSO‐*d*_*6*_), δ: 65.83 (CH_2_), 105.42, 115.35, 124.47, 125.66, 127.60, 128.24, 130.95, 133.92, 137.22, 141.94, 149.36, 159.59, 162.77, 168.95. APCI (m/z): [M+H]^+^: Theoretical value for C_18_H_14_ClN_3_O_3_S: 388.84/2, experimental value: 194.2.

##### 4.2.2.9 2‐[4‐[(2‐(4‐(4‐(3,4‐Dichlorophenyl)thiazol‐2‐yl))hydrazono)methyl]phenoxy]acetic acid (**2i**)

Yield: 71%. M.P.: 238°C–240°C. IR *ν* (cm^−1^): 3663 (O‐H), 2972, 2900 (Aliphatic C‐H), 1605–1423 (C=C, C=N), 1306–1057 (C‐O, C‐N), 872–696 (benzene ring out‐of‐plane deformation band). ^1^H‐NMR (300 MHz, DMSO‐*d*_*6*_), δ: 4.74 (s, 2H, CH_2_), 6.94 (d, 2H, phenoxy C2,6‐H), 7.52 (s, 1H, thiazole C5‐H), 7.60 (d, 2H, J: 8.14 Hz, phenoxy C3,5‐H), 7.66 (d, 1H, J: 8.43 Hz, phenyl C5‐H), 7.83 (d, 1H, J: 8.37 Hz, phenyl C6‐H), 8.00 (s, 1H, CH=N), 8.08 (s, 1H, phenyl C2‐H), 12.11 (NH). ^13^C‐NMR (75 MHz, DMSO‐*d*_*6*_), δ: 64.97 (CH_2_), 106.13, 115.13, 115.35, 126.00, 127.60, 127.71, 127.86, 128.25, 129.29, 130.10, 131.31, 131.88, 135.73, 141.98, 142.51, 148.38, 159.24, 159.60, 169.02, 170.44, 178.12. APCI (m/z): [M+H]^+^: Theoretical value for C_18_H_13_Cl_2_N_3_O_3_S: 423.29, experimental value: 423.6.

##### 4.2.2.10 2‐[4‐[(2‐(4‐(4‐([1,1′‐Biphenyl]‐4‐yl)thiazol‐2‐yl))hydrazono)methyl]phenoxy]acetic acid (**2j**)

Yield: 74%. M.P.: 215°C–218°C. IR *ν* (cm^−1^): 3275 (N‐H), 3109 (Aromatic C‐H), 2897 (Aliphatic C‐H), 1604–1406 (C=C, C=N), 1253–1170 (C‐O, C‐N), 831–731 (benzene ring out‐of‐plane deformation band). ^1^H‐NMR (300 MHz, DMSO‐*d*_*6*_), δ: 4.69 (s, 2H, CH_2_), 6.98 (d, 2H, J: 7.52 Hz, phenoxy C2,6‐H), 7.37–7.39 (m, 2H, Ar‐H), 7.47 (t, 2H, J: 7.15 Hz, Ar‐H), 7.59 (d, 2H, J: 7.95 Hz, Ar‐H), 7.71–7.72 (m, 4H, Ar‐H), 7.95 (d, 2H, J: 7.55 Hz, Ar‐H), 8.05 (s, 1H, CH=N), 12.12 (NH). ^13^C‐NMR (75 MHz, DMSO‐*d*_*6*_), δ: 65.37 (CH_2_), 104.09, 115.36, 126.55, 126.93, 127.27, 128.14, 129.43, 134.33, 139.44, 140.12, 141.68, 150.59, 159.31, 168.84, 170.54. APCI (m/z): [M+H]^+^: Theoretical value for C_24_H_19_N_3_O_3_S: 430.49, experimental value: 430.5.

##### 4.2.2.11 2‐[4‐[(2‐(4‐(4‐(4‐(4‐Fluorophenyl)thiazol‐2‐yl)))hydrazono)methyl]phenoxy]acetic acid (**2k**)

Yield: 67%. M.P.: 258°C–259°C. IR *ν* (cm^−1^): 3666 (O‐H), 2972, 2900 (Aliphatic C‐H), 1608–1408 (C=C, C=N), 1307–1055 (C‐O, C‐N), 825–684 (benzene ring out‐of‐plane deformation band). ^1^H‐NMR (300 MHz, DMSO‐*d*_*6*_), δ: 4.73 (s, 2H, CH_2_), 6.97 (d, 2H, J: 9.06 Hz, phenoxy C2,6‐H), 7.20–7.28 (m, 2H, phenyl C3,5‐H), 7.29 (s, 1H, thiazole C5‐H), 7.58 (d, 2H, J: 8.68 Hz, phenoxy C3,5‐H), 7.85 (dd, 2H, J: 8.94, 3.40 Hz, phenyl C2,6‐H), 7.98 (s, 1H, CH=N), 12.15 (NH). ^13^C‐NMR (75 MHz, DMSO‐*d*_*6*_), δ: 64.90 (CH_2_), 103.62, 115.31, 116.05, 127.91, 127.97, 128.18, 131.82, 141.62, 149.89, 159.15, 160.40, 163.64, 168.86, 170.48. APCI (m/z): [M+H]^+^: Theoretical value for C_18_H_14_FN_3_O_3_S: 372.34, experimental value: 372.5.

##### 4.2.2.12 2‐[4‐[(2‐(4‐(4‐(Naphthalen‐2‐yl)thiazol‐2‐yl))hydrazono)methyl]phenoxy]acetic acid (**2l**)

Yield: 68%. M.P.: 262°C–264°C. IR *ν* (cm^−1^): 3130 (N‐H), 1602–1506 (C=C, C=N), 1302–1053 (C‐O, C‐N), 925–713 (benzene ring out‐of‐plane deformation band). ^1^H‐NMR (300 MHz, DMSO‐*d*_*6*_), δ: 4.74 (s, 2H, CH_2_), 6.99 (d, 2H, J: 8.76 Hz, phenoxy C2,6‐H), 7.46–7.59 (m, 3H, Ar‐H), 7.61 (d, 2H, J: 7.88 Hz, Ar‐H), 7.89–8.00 (m, 5H, Ar‐H and CH=N), 8.37 (brs, 1H, Ar‐H), 12.37 (NH). ^13^C‐NMR (75 MHz, DMSO‐*d*_*6*_), δ: 64.91 (CH_2_), 104.72, 115.33, 124.40, 126.46, 126.90, 127.93, 128.04, 128.21, 132.60, 132.87, 133.62, 141.66, 150.84, 159.16, 168.85, 170.50. APCI (m/z): [M+H]^+^: Theoretical value for C_22_H_17_N_3_O_3_S: 404.45, experimental value: 404.4.

### 4.3 Biochemistry

For biological activity testing of the synthesized hydrazoyl thiazole derivatives, the tetrazole derivative known as MTT (3‐(4,5‐dimethylthiazol‐2‐yl)‐2,5‐diphenyltetrazolium bromide) was used to evaluate the metabolic activity of cells. To prepare a suitable culture medium in a 96‐well plate, trypsin is used to separate the cells from the surface. Subsequently, these cells are centrifuged. Trypan blue is added to the resulting suspension, and the live cells are counted under a microscope. The cell density was determined through calculation, and the cell suspension was adjusted to the desired density by adding culture medium. The cells in the prepared plate were seeded for 24 h at 37°C in a medium containing 5% CO_2_. Following the passaging process, the synthesized compounds were prepared in different concentrations using DMSO and incubated into the wells.

#### 4.3.1 MTT Assay

The assay was carried out as previously explained study (Mosmann 1983), with minor modifications. In summary, A549 lung adenocarcinoma cells, MCF‐7 breast cancer, and L929 embryonic mouse fibroblast cells were cultured in flasks containing Dulbecco's Modified Eagle's Medium and 10% fetal bovine serum (FBS) in a CO_2_ incubator at 37°C with 5% CO_2_. Exponentially growing cells were plated at 2 × 10^4^ cells/mL into 96‐well cell culture microplates and incubated for 24 h before the addition of the compounds. The stock solutions of the compounds were prepared in dimethyl sulfoxide (DMSO) and further dilutions were made with a fresh culture medium. The concentration of DMSO in the final culture medium was 0.1% which had no effect on cell viability. After preincubation, the compounds were added to give a final concentration in the range of 1–1000 μM and the cells were incubated at 37°C. When the incubation time reached 24 h, 20 μL of 3‐(4,5‐dimethylthiazol‐2‐yl)‐2,5‐diphenyltetrazolium bromide (MTT, Sigma‐Aldrich, St. Louis, MO, USA) was added to each well (final concentration 0.5 mg/mL) and the cells were incubated in a humidified atmosphere for 2–4 h. Then, MTT dye was removed from the cells, and 200 μL of DMSO was added to each well, followed by a 10‐min incubation. The color change was determined at a wavelength of 540 nm with the microplate spectrophotometer (Bio‐Tek, Winooski, VT, USA). Half maximal inhibitory concentration (IC_50_) data (μM) were determined using Microsoft Excel by plotting percentage cell viability against the log‐transformed compound concentrations (log_10_) and calculating the concentration required for 50% cell growth inhibition via the linear regression equation derived from the dose–response curve. IC_50_ values expressed as mean ± SD. Cisplatin was used as the positive control. All the experiments were triplicated.

$$\begin{array}{c} \%\text{Inhibition}=\\ 100-\left[\left({\mathrm{OD}}_{\text{test substance}}-{\mathrm{OD}}_{\text{blank}}/{\mathrm{OD}}_{\text{solvent control}}-{\mathrm{OD}}_{\text{blank}}\right)\times 100\right] \end{array}$$

#### 4.3.2 Annexin V‐FITC/PI Flow Cytometry Method

In the Annexin V‐FITC/PI method (Çiftçi et al. 2015; Kaya et al. 2025), A549 lung cancer cells and MCF‐7 breast cancer cells were cultured into 6‐well plates at a concentration of 2 × 10^5^ cells/ml per well, and then incubated for 24 h. After 24 h, cisplatin, designated as the control drug, and the synthesized molecules (compounds **2b**, **2c**, **2e**, **2f**, **2k**, **2l**), were treated with to the wells at IC_50_ concentrations and incubated again for 24 h. Then, the cells were washed using phosphate‐buffered saline (PBS) and treated with trypsin and transferred to suitable tubes for flow cytometry. The centrifuge was set to 1200 rpm and centrifuged for 4 min. Afterwards centrifugation, the supernatant was discarded from the tube, and 1 mL of PBS was added to the resulting cell pellet to wash it. Then, A549 and MCF‐7 cells were resuspended in 100 μL Binding Buffer. Cells were stained with 5 μL of Annexin V‐FITC and 5 μL of Propidium Iodide (PI) for 15 min at room temperature in the dark. After incubation, 400 5 μL Binding Buffer was added to each tube. Apoptosis was subsequently measured using FITC Annexin V Apoptosis Detection Kit, according to the manufacturer's instructions (BD Biosciences, San Jose, CA, USA). Data acquisition was performed using a Beckman Coulter CytoFLEX (Indianapolis, USA) flow cytometer. Fluorescence signals were excited using a 488 nm blue laser; Annexin V‐FITC emission was collected using a 530/30 nm filter (FL1/FITC), and PI emission was collected using a 610/20 nm (veya 585/42 nm) filter (FL2/PI). A total of 10.000 events (cells) per sample were acquired. Data analysis and gating were conducted using CytExpert software. Cells were first gated on Forward Scatter to exclude doublets, and Side Scatter (SSC‐A vs. FSC‐A) to isolate intact single‐cell populations from debris. Quadrant gates were defined based on unstained controls for spectral compensation. Cell populations were classified into four quadrants: Q4/Lower‐Left (FITC^−^/PI^−^): Viable cells. Q3/Lower‐Right (FITC^+^/PI^−^): Early apoptotic cells. Q2/Upper‐Right (FITC^+^/PI^+^): Late apoptotic cells. Q1/Upper‐Left (FITC^−^/PI^+^): Necrotic cells.

#### 4.3.3 Caspase‐3 Activation Procedure

In the caspase 3 activation method (Çiftçi et al. 2015; Kaya et al. 2025), A549 lung cancer cells and MCF‐7 breast cancer cells were cultured into 6‐well plates at a concentration of 2 × 10^5^ cells/ml per well, and then incubated for 24 h. After 24 h, cisplatin, designated as the control drug, and the synthesized molecules (compounds **2b**, **2c**, **2d**, **2e**, **2f**, **2k**, **2l**), were treated with the wells at IC_50_ concentrations and incubated again for 24 h. Then, the cells were washed using phosphate‐buffered saline (PBS) and treated with trypsin and transferred to suitable tubes for flow cytometry. The centrifuge was set to 1200 rpm and centrifuged for 4 min. Afterwards centrifugation, the supernatant was discarded from the tube, and 1 mL of PBS was added to the resulting cell pellet to wash it. Then, the caspase‐3 activity measurement protocol was performed according to the manufacturer's instructions (BD Biosciences Pharmingen). The cells were briefly washed with cold PBS and incubated with 0.5 mL of permeabilizing lysis solution for 30 min at room temperature in the dark. The pellets were washed twice with 0.5 mL of permeabilizing wash buffer. The cells were suspended in 100 μL of permeabilizing wash buffer, and 10 μL of caspase‐3 antibody was added; the mixture was then incubated for 20 min at room temperature in the dark. At least 10,000 cells were counted for each sample, and the cells were analyzed using a Beckman Coulter CytoFLEX (Indianapolis, USA) flow cytometer using CytExpert software.

### 4.4 In Silico Studies

#### 4.4.1 Molecular Docking

Within the scope of the study, all the final compounds' carboxylate anion forms were generated using Avogadro (Version 1.103.0) (Hanwell and Hutchison 2026), and the structures were optimized by energy minimization employing the MMFF94 force field. The optimized molecules were then prepared and saved in PDBQT format using AutoDockTools (v. 1.5.7) (The Scripps Research Institute 2017).

The protein structure (PDB ID: 4QTX) was retrieved from the RCSB Protein Data Bank (rcsb.org) and prepared using AutoDockTools. All hydrogen atoms were added, and non‐polar hydrogens were merged. Gasteiger partial charges were assigned to the protein, after which the prepared structure was exported and saved in PDBQT format.

The docking grid box was centered at X = 107.40, Y = 95.474, Z = 23.194, with the grid dimensions set to 20 × 20 × 20 Å, and the exhaustiveness parameter was set to 64. Molecular docking was performed using AutoDock Vina (v. 1.2.0) (Eberhardt et al. 2021; Trott and Olson 2010) for all final compounds.

#### 4.4.2 Molecular Dynamics Simulation

Based on the binding poses obtained from molecular docking with the 4QTX structure, 100 ns molecular dynamics simulations were performed using GROMACS (GROMACS Development Team 2025; Abraham et al. 2015).

The protein was parameterized using the CHARMM force field (June 2022 release), while ligand parameters were generated with the CHARMM General Force Field (CGenFF) using the CGenFF program/web server (Vanommeslaeghe et al. 2010). A simulation box was constructed around the complex and filled with TIP3P water molecules. To neutralize the system, Na^+^ and Cl^−^ ions were added to achieve a final concentration of 0.15 M.

Energy minimization was carried out stepwise in four stages. In the first three stages, positional restraints were applied to the entire protein and ligand (excluding hydrogen atoms) with force constants of 41,840, 4184, and 1000 kJ mol^−1^ nm^−2^, respectively, in all directions. No restraints were applied in the final stage. Each minimization step was terminated when the maximum force dropped below 10 kJ mol^−1^, and an emstep value of 0.001 was used in all stages.

A 400 ps heating protocol was subsequently applied. The system was heated from 50 K to 300 K over the first 200 ps, and the temperature was then maintained at 300 K until 400 ps. During this step, positional restraints of 1000 kJ·mol^−1^·nm^−2^ were applied to the protein and ligand (excluding hydrogen atoms). Temperature coupling was performed using the v‐rescale thermostat.

Pressure equilibration was performed in three consecutive stages. In the first stage, the protein and ligand (excluding hydrogen atoms) were restrained with a force constant of 1000 kJ·mol^−1^·nm^−2^; in the second stage, the restraint strength was reduced to 250 kJ·mol^−1^·nm^−2^. No restraints were applied in the third stage. Each stage lasted 5 ns, resulting in a total equilibration time of 6 ns. The v‐rescale thermostat was used throughout, while pressure coupling was maintained with the c‐rescale barostat in the first two stages and the Parrinello–Rahman barostat in the final stage.

A 100 ns molecular dynamics simulation was performed without applying any restraints. Temperature coupling was kept using the v‐rescale thermostat, and pressure coupling was checked using the Parrinello–Rahman barostat.

For all simulation steps, the PME order was set to 4 and the Coulomb cutoff radius (*r*_coulomb_) was set to 1.2 nm. The van der Waals interactions were treated using the *Switch* scheme: vdW interactions were computed fully up to 1.0 nm, smoothly switched off between 1.0 and 1.2 nm, and not calculated beyond 1.2 nm. Hydrogen‐bond constraints were applied using the LINCS algorithm.

The trajectories obtained from the molecular dynamics simulations were analyzed and visualized using GROMACS. Specifically, the time‐dependent RMSD of the isomers, the time‐dependent RMSF of the chain to which each isomer binds, and the buried surface area (BSA) between the isomers and the protein as a function of time were calculated and plotted.

The time‐dependent interactions between the isomers and the enzyme—including interaction types and their occurrence percentages—were calculated and visualized using ProLIF (v2.0.3) (Bouysset 2024; Bouysset and Fiorucci 2021).

The simulation trajectories were recorded as videos using Visual Molecular Dynamics (v1.9.4) (University of Illinois at Urbana–Champaign, Theoretical and Computational Biophysics Group 2020; Humphrey et al. 1996). In these visualizations, water molecules and ions were omitted, the protein was represented in ribbon form, and the ligand was rendered in an enlarged representation to enhance clarity.

#### 4.4.3 DFT Studies

The energy‐minimized carboxylate anion structures obtained in the ligand preparation step were subsequently saved in XYZ format for use in the DFT calculations.

DFT calculations for all final compounds were performed using Gaussian 16 (Frisch et al. 2016), and molecular visualizations were enforced with GaussView 6 (Dennington et al. 2016). Geometry optimizations and frequency calculations were executed at the B3LYP/6‐31+G(d,p) level of theory using the options Opt=(CalcFC, MaxStep=5), Freq, Int=UltraFine, SCF=(XQC, Tight), and NoSymm. Solvent effects were taken into account using the SMD implicit solvation model with water as the solvent via SCRF=(SMD, Solvent=Water). The optimized geometries were confirmed as true minima by the absence of imaginary frequencies. In addition, HOMO and LUMO energies, including the HOMO–LUMO energy gaps, were obtained.

To further investigate local reactivity, single‐point calculations were performed on the optimized geometries of the carboxylate forms at the wB97XD/aug‐cc‐pVDZ level in aqueous solution using the SMD solvation model. Hirshfeld population analysis was carried out for the *N*, *N* − 1, and *N* + 1 electron states, corresponding to the monoanionic, neutral radical, and radical dianionic species, respectively. The resulting atomic charge distributions were then used to derive the condensed Fukui functions. The condensed Fukui functions were calculated according to the equations given below.

$$f_{k}^{+}=q_{k}\left(N\right)-q_{k}\left(N+1\right)$$

$$f_{k}^{-}=q_{k}\left(N-1\right)-q_{k}\left(N\right)$$

$$f_{k}^{0}=\frac{q_{k}\left(N-1\right)-q_{k}\;\left(N+1\right)\;}{2}$$

In these expressions, $q_{k}$ represents the Hirshfeld atomic charge on atom k, and N, N−1, and N+1 correspond to the monoanionic, neutral radical, and radical dianionic states, respectively. Thus, $f_{k}^{+}$, $f_{k}^{-}$, and $f_{k}^{0}$ refer to the condensed Fukui functions for nucleophilic, electrophilic, and radical attack, respectively.

## Author Contributions

**Erol Akgün:** conceptualization, software, visualization. **Leyla Yurttaş:** conceptualization, methodology, investigation, writing – review and editing, writing – original draft, project administration, supervision. **Zeynep Zişan Demirci:** conceptualization, methodology, writing – original draft. **Gülşen Akalın Çiftçi:** investigation, writing – review and editing, methodology.

## Funding

This study is supported by Anadolu University.

## Conflicts of Interest

The authors declare no conflicts of interest.

## Supporting information


**Supporting Information: S1:** **Figure 1**. IR spectrum of compound 2a.
**Figure 2**. ^1^H‐NMR spectrum of compound 2a.
**Figure 3**. ^13^C‐NMR spectrum of compound 2a.
**Figure 4**. LC–MS spectrum of compound 2a.
**Figure 5**. IR spectrum of compound 2b.
**Figure 6**. ^1^H‐NMR spectrum of compound 2b.
**Figure 7**. ^13^C‐NMR spectrum of compound 2b.
**Figure 8**. LC–MS spectrum of compound 2b.
**Figure 9**. IR spectrum of compound 2c.
**Figure 10**. ^1^H‐NMR spectrum of compound 2c.
**Figure 11**. ^13^C‐NMR spectrum of compound 2c.
**Figure 12**. LC–MS spectrum of compound 2c.
**Figure 13**. IR spectrum of compound 2d.
**Figure 14**. ^1^H‐NMR spectrum of compound 2d.
**Figure 15**. ^13^C‐NMR spectrum of compound 2d.
**Figure 16**. LC–MS spectrum of compound 2d.
**Figure 17**. IR spectrum of compound 2e.
**Figure 18**. ^1^H‐NMR spectrum of compound 2e.
**Figure 19**. ^13^C‐NMR spectrum of compound 2e.
**Figure 20**. LC–MS spectrum of compound 2e.
**Figure 21**. IR spectrum of compound 2f.
**Figure 22**. ^1^H‐NMR spectrum of compound 2f.
**Figure 23**. ^13^C‐NMR spectrum of compound 2f.
**Figure 24**. LC–MS spectrum of compound 2f.
**Figure 25**. IR spectrum of compound 2g.
**Figure 26**. ^1^H‐NMR spectrum of compound 2g.
**Figure 27**. ^13^C‐NMR spectrum of compound 2g.
**Figure 28**. LC–MS spectrum of compound 2g.
**Figure 29**. IR spectrum of compound 2h.
**Figure 30**. ^1^H‐NMR spectrum of compound 2h.
**Figure 31**. ^13^C‐NMR spectrum of compound 2h.
**Figure 32**. LC–MS spectrum of compound 2h.
**Figure 33**. IR spectrum of compound 2i.
**Figure 34**. ^1^H‐NMR spectrum of compound 2i.
**Figure 35**. ^13^C‐NMR spectrum of compound 2i.
**Figure 36**. LC–MS spectrum of compound 2i.
**Figure 37**. IR spectrum of compound 2j.
**Figure 38**. ^1^H‐NMR spectrum of compound 2j.
**Figure 39**. ^13^C‐NMR spectrum of compound 2j.
**Figure 40**. LC–MS spectrum of compound 2j.
**Figure 41**. IR spectrum of compound 2k.
**Figure 42**. ^1^H‐NMR spectrum of compound 2k.
**Figure 43**. ^13^C‐NMR spectrum of compound 2k.
**Figure 44**. LC–MS spectrum of compound 2k.
**Figure 45**. IR spectrum of compound 2l.
**Figure 46**. ^1^H‐NMR spectrum of compound 2l.
**Figure 47**. ^13^C‐NMR spectrum of compound 2l.
**Figure 48**. LC–MS spectrum of compound 2l.
**Figure 49**. Dose–response graphs of the compounds against A549 cell line.
**Figure 50**. Dose–response graphs of the compounds against MCF‐7 cell line.
**Figure 51**. Dose–response graphs of the compounds against L929 cell line.

**Supporting Information: S1.** **Figure 1**. Frontier molecular orbitals (HOMO, left; LUMO, right) of compound 2a.
**Figure 2**. Frontier molecular orbitals (HOMO, left; LUMO, right) of compound 2b.
**Figure 3**. Frontier molecular orbitals (HOMO, left; LUMO, right) of compound 2c.
**Figure 4**. Frontier molecular orbitals (HOMO, left; LUMO, right) of compound 2d.
**Figure 5**. Frontier molecular orbitals (HOMO, left; LUMO, right) of compound 2e.
**Figure 6**. Frontier molecular orbitals (HOMO, left; LUMO, right) of compound 2f.
**Figure 7**. Frontier molecular orbitals (HOMO, left; LUMO, right) of compound 2g.
**Figure 8**. Frontier molecular orbitals (HOMO, left; LUMO, right) of compound 2h.
**Figure 9**. Frontier molecular orbitals (HOMO, left; LUMO, right) of compound 2i.
**Figure 10**. Frontier molecular orbitals (HOMO, left; LUMO, right) of compound 2j.
**Figure 11**. Frontier molecular orbitals (HOMO, left; LUMO, right) of compound 2k.
**Figure 12**. Frontier molecular orbitals (HOMO, left; LUMO, right) of compound 2l.
**Figure 13**. Atom numbering of compound 2a used for Hirshfeld charge and condensed Fukui function analyses.
**Figure 14**. Atom numbering of compound 2b used for Hirshfeld charge and condensed Fukui function analyses.
**Figure 15**. Atom numbering of compound 2c used for Hirshfeld charge and condensed Fukui function analyses.
**Figure 16**. Atom numbering of compound 2d used for Hirshfeld charge and condensed Fukui function analyses.
**Figure 17**. Atom numbering of compound 2e used for Hirshfeld charge and condensed Fukui function analyses.
**Figure 18**. Atom numbering of compound 2f used for Hirshfeld charge and condensed Fukui function analyses.
**Figure 19**. Atom numbering of compound 2g used for Hirshfeld charge and condensed Fukui function analyses.
**Figure 20**. Atom numbering of compound 2h used for Hirshfeld charge and condensed Fukui function analyses.
**Figure 21**. Atom numbering of compound 2i used for Hirshfeld charge and condensed Fukui function analyses.
**Figure 22**. Atom numbering of compound 2j used for Hirshfeld charge and condensed Fukui function analyses.
**Figure 23**. Atom numbering of compound 2k used for Hirshfeld charge and condensed Fukui function analyses.
**Figure 24**. Atom numbering of compound 2l used for Hirshfeld charge and condensed Fukui function analyses.
**Table 1**. Hirshfeld atomic charges and condensed Fukui functions (f^+^, f^−^, f^0^) for compound 2a.
**Table 2**. Hirshfeld atomic charges and condensed Fukui functions (f^+^, f^−^, f^0^) for compound 2b.
**Table 3**. Hirshfeld atomic charges and condensed Fukui functions (f^+^, f^−^, f^0^) for compound 2c.
**Table 4**. Hirshfeld atomic charges and condensed Fukui functions (f^+^, f^−^, f^0^) for compound 2d.
**Table 5**. Hirshfeld atomic charges and condensed Fukui functions (f^+^, f^−^, f^0^) for compound 2e.
**Table 6**. Hirshfeld atomic charges and condensed Fukui functions (f^+^, f^−^, f^0^) for compound 2f.
**Table 7**. Hirshfeld atomic charges and condensed Fukui functions (f^+^, f^−^, f^0^) for compound 2g.
**Table 8**. Hirshfeld atomic charges and condensed Fukui functions (f^+^, f^−^, f^0^) for compound 2h.
**Table 9**. Hirshfeld atomic charges and condensed Fukui functions (f^+^, f^−^, f^0^) for compound 2i.
**Table 10**. Hirshfeld atomic charges and condensed Fukui functions (f^+^, f^−^, f^0^) for compound 2j.
**Table 11**. Hirshfeld atomic charges and condensed Fukui functions (f^+^, f^−^, f^0^) for compound 2k.
**Table 12**. Hirshfeld atomic charges and condensed Fukui functions (f^+^, f^−^, f^0^) for compound 2l.

## Data Availability

All the results obtained in this study are presented in manuscript and Supporting Information and it was presented as a master's thesis in 2025.

## References

[cbdd70387-bib-0001] Abraham, M. J. , T. Murtola , R. Schulz , et al. 2015. “GROMACS: High Performance Molecular Simulations Through Multi‐Level Parallelism From Laptops to Supercomputers.” SoftwareX 1–2: 19–25.

[cbdd70387-bib-0002] Ayati, A. , S. Emami , A. Asadipour , A. Shafiee , and A. Foroumadi . 2015. “Recent Applications of 1,3‐Thiazole Core Structure in the Identification of New Lead Compounds and Drug Discovery.” European Journal of Medicinal Chemistry 97: 699–718. 10.1016/j.ejmech.2015.04.015. 25934508

[cbdd70387-bib-0003] Bouysset, C. 2024. ProLIF (Version 2.0.3) [Computer Software]. Zenodo.

[cbdd70387-bib-0004] Bouysset, C. , and S. Fiorucci . 2021. “ProLIF: A Library to Encode Molecular Interactions as Fingerprints.” Journal of Cheminformatics 13: 72. 34563256 10.1186/s13321-021-00548-6 PMC8466659

[cbdd70387-bib-0005] Bray, F. , M. Laversanne , H. Sung , et al. 2024. “Global Cancer Statistics 2022: GLOBOCAN Estimates of Incidence and Mortality Worldwide for 36 Cancers in 185 Countries.” CA: A Cancer Journal for Clinicians 74: 229–263. 10.3322/caac.21834. 38572751

[cbdd70387-bib-0006] Carter, J. S. , S. Kramer , J. J. Talley , et al. 1999. “Synthesıs and Activity of Sulfonamide‐Substituted 4,5‐Diaryl Thiazoles as Selective Cyclooxygenase‐2 Inhibitors.” Bioorganic & Medicinal Chemistry Letters 9: 1171–1174. 10328307 10.1016/s0960-894x(99)00157-2

[cbdd70387-bib-0007] Cheng, S. , K. Swanson , I. Eliaz , J. N. Mcclintick , G. E. Sandusky , and D. Sliva . 2015. “Pachymic Acid Inhibits Growth and Induces Apoptosis of Pancreatic Cancer In Vitro and In Vivo by Targeting ER Stress.” PLoS One 10: e0122270. 10.1371/journal.pone.0122270. 25915041 PMC4411097

[cbdd70387-bib-0008] Çiftçi, G. A. , A. Işcan , and M. Kutlu . 2015. “Escin Reduces Cell Proliferation and Induces Apoptosis on Glioma and Lung Adenocarcinoma Cell Lines.” Cytotechnogy 67, no. 5: 893–904. 10.1007/s10616-015-9877-6. PMC4545433 25906387

[cbdd70387-bib-0009] Dennington, R. , T. A. Keith , and J. M. Millam . 2016. GaussView, Version 6. Semichem Inc.

[cbdd70387-bib-0010] Eberhardt, J. , D. Santos‐Martins , A. F. Tillack , and S. Forli . 2021. “AutoDock Vina 1.2.0: New Docking Methods, Expanded Force Field, and Python Bindings.” Journal of Chemical Information and Modeling 61, no. 8: 3891–3898. 34278794 10.1021/acs.jcim.1c00203 PMC10683950

[cbdd70387-bib-0011] Elmore, S. 2007. “Apoptosis: A Review of Programmed Cell Death.” Toxicologic Pathology 35, no. 4: 495–516. 10.1080/01926230701320337. 17562483 PMC2117903

[cbdd70387-bib-0012] Evren, A. E. , D. Nuha , S. Dawbaa , A. B. Karaduman , and L. Yurttaş . 2024. “Novel Thiazole‐Hydrazide Derivatives and Their Anticancer Properties.” Clinical and Experimental Health Sciences 14, no. 3: 783–789. 10.33808/clinexphealthsci.1414252.

[cbdd70387-bib-0013] Farghaly, T. A. , G. S. Masaret , Z. A. Muhammad , and M. F. Harras . 2020. “Discovery of Thiazole‐Based‐Chalcones and 4‐Hetarylthiazoles as Potent Anticancer Agents: Synthesis, Docking Study and Anticancer Activity.” Bioorganic Chemistry 98: 103761. 10.1016/J.BIOORG.2020.103761. 32200332

[cbdd70387-bib-0014] Frisch, M. J. , G. W. Trucks , H. B. Schlegel , et al. 2016. Gaussian 16, Revision C.01. Gaussian, Inc.

[cbdd70387-bib-0015] GROMACS Development Team . 2025. GROMACS (Version 2025.2) [Computer Software]. Zenodo.

[cbdd70387-bib-0016] Hanwell, M. , and G. Hutchison . 2026. Avogadro(Version 1.103.0) [Computer Software]. Zenodo.

[cbdd70387-bib-0017] Humphrey, W. , A. Dalke , and K. Schulten . 1996. “VMD: Visual Molecular Dynamics.” Journal of Molecular Graphics 14, no. 1: 33–38. 8744570 10.1016/0263-7855(96)00018-5

[cbdd70387-bib-0018] Kaya, A. Z. , A. E. Evren , H. E. Temel , G. A. Çiftçi , and L. Yurttaş . 2025. “Novel Thiazole Based 4‐Nitrosalicyl Hydrazone Derivatives as Anticancer Agents.” Journal of Molecular Structure 1348: 143486. 10.1016/j.molstruc.2025.143486.

[cbdd70387-bib-0019] Lvov, A. G. , M. M. Khusniyarov , and V. Z. Shirinian . 2018. “Azole‐Based Diarylethenes as the Next Step Towards Advanced Photochromic Materials.” Journal of Photochemistry and Photobiology C: Photochemistry Reviews 36: 1–23. 10.1016/J.JPHOTOCHEMREV.2018.04.002.

[cbdd70387-bib-0020] Moslehi, M. , S. Rezaei , P. Talebzadeh , et al. 2023. “Apigenin in Cancer Therapy: Prevention of Genomic Instability and Anticancer Mechanisms.” Clinical and Experimental Pharmacology & Physiology 50: 3–18. 10.1111/1440-1681.13725. 36111951

[cbdd70387-bib-0021] Mosmann, T. 1983. “Rapid Colorimetric Assay for Cellular Growth and Survival: Application to Proliferation and Cytotoxicity Assays.” Journal of Immunological Methods 65, no. 1–2: 55–63. 10.1016/0022-1759(83)90303-4. 6606682

[cbdd70387-bib-0022] Nauen, R. , U. Ebbinghaus‐Kintscher , V. L. Salgado , and M. Kaussmann . 2003. “Thiamethoxam Is a Neonicotinoid Precursor Converted to Clothianidin in Insects and Plants.” Pesticide Biochemistry and Physiology 76: 55–69. 10.1016/S0048-3575(03)00065-8.

[cbdd70387-bib-0023] Orujova, T. , A. Ece , G. A. Çiftçi , A. Özdemir , and M. D. Altıntop . 2023. “A New Series of Thiazole‐Hydrazone Hybrids for Akt‐Targeted Therapy of Non‐Small Cell Lung Cancer.” Drug Development Research 84, no. 2: 185–199. 10.1002/ddr.22022. 36469421

[cbdd70387-bib-0024] Oukoloff, K. , B. Lucero , K. R. Francisco , K. R. Brunden , and C. Ballatore . 2019. “1,2,4‐Triazolo[1,5‐a]Pyrimidines in Drug Design.” European Journal of Medicinal Chemistry 165: 332–346. 10.1016/j.ejmech.2019.01.027. 30703745 PMC6394845

[cbdd70387-bib-0025] Pandya, S. R. , H. Singh , M. F. Desimone , J. Singh , N. George , and S. Jasani . 2023. “Circumventing Challenges in Mitochondrial Targeting for Cancer Treatment: Leveraging Nanoplatforms for Effective Solutions.” Materials Advances 5: 409–431. 10.1039/d3ma00629h.

[cbdd70387-bib-0026] Porter, A. G. , and R. U. Jänicke . 1999. “Emerging Roles of Caspase‐3 in Apoptosis.” Cell Death and Differentiation 6, no. 2: 99–104. 10.1038/sj.cdd.4400476. 10200555

[cbdd70387-bib-0027] Sever, B. , C. Türkeş , Y. Demir , et al. 2025. “Hydrazonylthiazole Derivatives as Dual EGFR and ALR2 Inhibitors: Design, Synthesis, and Comprehensive In Vitro and In Silico Evaluation for Potential Anticancer Activity.” Pharmaceuticals 19, no. 1: 50. 10.3390/ph19010050. 41599651 PMC12845312

[cbdd70387-bib-0028] Sharma, R. N. , F. P. Xavier , K. K. Vasu , S. C. Chaturvedi , and S. S. Pancholi . 2009. “Synthesis of 4‐Benzyl‐1,3‐Thiazole Derivatives as Potential Anti‐Inflammatory Agents: An Analogue‐Based Drug Design Approach.” Journal of Enzyme Inhibition and Medicinal Chemistry 24: 890–897. 10.1080/14756360802519558. 19469712

[cbdd70387-bib-0029] Sung, H. , J. Ferlay , R. L. Siegel , et al. 2021. “Global Cancer Statistics 2020: GLOBOCAN Estimates of Incidence and Mortality Worldwide for 36 Cancers in 185 Countries.” CA: A Cancer Journal for Clinicians 71: 209–249. 10.3322/caac.21660. 33538338

[cbdd70387-bib-0030] Tafere, D. A. , M. Gebrezgiabher , F. Elemo , et al. 2025. “Hydrazones, Hydrazones‐Based Coinage Metal Complexes, and Their Biological Applications.” RSC Advances 15, no. 8: 6191–6207. 10.1039/d4ra07794f. 40034805 PMC11873977

[cbdd70387-bib-0031] Takrouri, K. , T. Chen , E. Papadopoulos , et al. 2014. “Structure–Activity Relationship Study of 4EGI‐1, Small Molecule eIF4E/eIF4G Protein–Protein Interaction Inhibitors.” European Journal of Medicinal Chemistry 77: 361–377. 10.1016/j.ejmech.2014.03.034. 24675136 PMC4128255

[cbdd70387-bib-0032] The Scripps Research Institute . 2017. “AutoDockTools (Version 1.5.7) [Computer Software]”.

[cbdd70387-bib-0033] Trott, O. , and A. J. Olson . 2010. “AutoDock Vina: Improving the Speed and Accuracy of Docking With a New Scoring Function, Efficient Optimization, and Multithreading.” Journal of Computational Chemistry 31, no. 2: 455–461. 19499576 10.1002/jcc.21334 PMC3041641

[cbdd70387-bib-0034] University of Illinois at Urbana–Champaign, Theoretical and Computational Biophysics Group . 2020. “VMD (Version 1.9.4) [Computer Software].”

[cbdd70387-bib-0035] Vanommeslaeghe, K. , E. Hatcher , C. Acharya , et al. 2010. “CHARMM General Force Field (CGenFF): A Force Field for Drug‐Like Molecules Compatible With the CHARMM All‐Atom Additive Biological Force Fields.” Journal of Computational Chemistry 31, no. 4: 671–690. 19575467 10.1002/jcc.21367 PMC2888302

[cbdd70387-bib-0036] Wang, Y.‐T. , X. Huang , X.‐C. Cai , X.‐X. Kang , and H.‐L. Zhu . 2024. “Synthesis, Biological Evaluation and Molecular Docking of Thiazole Hydrazone Derivatives Grafted With Indole as Novel Tubulin Polymerization Inhibitors.” Journal of Molecular Structure 1301: 137343.

[cbdd70387-bib-0037] Yurttaş, L. , G. A. Çiftçi , and H. E. Temel . 2021. “Evaluation of New Sulfathiazole Derivatives as Antiproliferative Agents.” Turkish Journal of Biochemistry 46, no. 5: 533–539. 10.1515/tjb-2020-0238.

