# Supplementary material for: Investigation of the In Vitro and In Silico Anticancer Potential of Novel 4‐Arylthiazole‐Hydrazone Derivatives
Source: Chem Biol Drug Des. 2026 Aug 30;108(3):e70387. doi: 10.1111/cbdd.70387 (PMC13527178; doi:10.1111/cbdd.70387)
Supplement: Supplementary file 1 [file CBDD-108-e70387-s001.docx]

**Supplement Material 2**

**List of Figures**

[Figure 1. Frontier molecular orbitals (HOMO, left; LUMO, right) of compound 2a.	2](#_Toc223890808)

[Figure 2. Frontier molecular orbitals (HOMO, left; LUMO, right) of compound 2b.	2](#_Toc223890809)

[Figure 3. Frontier molecular orbitals (HOMO, left; LUMO, right) of compound 2c.	2](#_Toc223890810)

[Figure 4. Frontier molecular orbitals (HOMO, left; LUMO, right) of compound 2d.	2](#_Toc223890811)

[Figure 5. Frontier molecular orbitals (HOMO, left; LUMO, right) of compound 2e.	3](#_Toc223890812)

[Figure 6. Frontier molecular orbitals (HOMO, left; LUMO, right) of compound 2f.	3](#_Toc223890813)

[Figure 7. Frontier molecular orbitals (HOMO, left; LUMO, right) of compound 2g.	3](#_Toc223890814)

[Figure 8. Frontier molecular orbitals (HOMO, left; LUMO, right) of compound 2h.	3](#_Toc223890815)

[Figure 9. Frontier molecular orbitals (HOMO, left; LUMO, right) of compound 2i.	3](#_Toc223890816)

[Figure 10. Frontier molecular orbitals (HOMO, left; LUMO, right) of compound 2j.	4](#_Toc223890817)

[Figure 11. Frontier molecular orbitals (HOMO, left; LUMO, right) of compound 2k.	4](#_Toc223890818)

[Figure 12. Frontier molecular orbitals (HOMO, left; LUMO, right) of compound 2l.	4](#_Toc223890819)

[Figure 13. Atom numbering of compound 2a used for Hirshfeld charge and condensed Fukui function analyses.	5](#_Toc223890820)

[Figure 14. Atom numbering of compound 2b used for Hirshfeld charge and condensed Fukui function analyses.	6](#_Toc223890821)

[Figure 15. Atom numbering of compound 2c used for Hirshfeld charge and condensed Fukui function analyses.	7](#_Toc223890822)

[Figure 16. Atom numbering of compound 2d used for Hirshfeld charge and condensed Fukui function analyses.	8](#_Toc223890823)

[Figure 17. Atom numbering of compound 2e used for Hirshfeld charge and condensed Fukui function analyses.	9](#_Toc223890824)

[Figure 18. Atom numbering of compound 2f used for Hirshfeld charge and condensed Fukui function analyses.	10](#_Toc223890825)

[Figure 19. Atom numbering of compound 2g used for Hirshfeld charge and condensed Fukui function analyses.	11](#_Toc223890826)

[Figure 20. Atom numbering of compound 2h used for Hirshfeld charge and condensed Fukui function analyses.	12](#_Toc223890827)

[Figure 21. Atom numbering of compound 2i used for Hirshfeld charge and condensed Fukui function analyses.	13](#_Toc223890828)

[Figure 22. Atom numbering of compound 2j used for Hirshfeld charge and condensed Fukui function analyses.	14](#_Toc223890829)

[Figure 23. Atom numbering of compound 2k used for Hirshfeld charge and condensed Fukui function analyses.	15](#_Toc223890830)

[Figure 24. Atom numbering of compound 2l used for Hirshfeld charge and condensed Fukui function analyses.	16](#_Toc223890831)

**List of Tables**

[Table 1. Hirshfeld atomic charges and condensed Fukui functions (f⁺, f⁻, f⁰) for compound 2a.ᵃ	5](#_Toc223301285)

[Table 2. Hirshfeld atomic charges and condensed Fukui functions (f⁺, f⁻, f⁰) for compound 2b.*ᵃ*	6](#_Toc223301286)

[Table 3. Hirshfeld atomic charges and condensed Fukui functions (f⁺, f⁻, f⁰) for compound 2c.*ᵃ*	7](#_Toc223301287)

[Table 4. Hirshfeld atomic charges and condensed Fukui functions (f⁺, f⁻, f⁰) for compound 2d.*ᵃ*	8](#_Toc223301288)

[Table 5. Hirshfeld atomic charges and condensed Fukui functions (f⁺, f⁻, f⁰) for compound 2e.*ᵃ*	9](#_Toc223301289)

[Table 6. Hirshfeld atomic charges and condensed Fukui functions (f⁺, f⁻, f⁰) for compound 2f.*ᵃ*	10](#_Toc223301290)

[Table 7. Hirshfeld atomic charges and condensed Fukui functions (f⁺, f⁻, f⁰) for compound 2g.*ᵃ*	11](#_Toc223301291)

[Table 8. Hirshfeld atomic charges and condensed Fukui functions (f⁺, f⁻, f⁰) for compound 2h.*ᵃ*	12](#_Toc223301292)

[Table 9. Hirshfeld atomic charges and condensed Fukui functions (f⁺, f⁻, f⁰) for compound 2i.*ᵃ*	13](#_Toc223301293)

[Table 10. Hirshfeld atomic charges and condensed Fukui functions (f⁺, f⁻, f⁰) for compound 2j.*ᵃ*	14](#_Toc223301294)

[Table 11. Hirshfeld atomic charges and condensed Fukui functions (f⁺, f⁻, f⁰) for compound 2k.*ᵃ*	15](#_Toc223301295)

[Table 12. Hirshfeld atomic charges and condensed Fukui functions (f⁺, f⁻, f⁰) for compound 2l.*ᵃ*	16](#_Toc223301296)

Figure 1. Frontier molecular orbitals (HOMO, left; LUMO, right) of compound 2a.

Figure 2. Frontier molecular orbitals (HOMO, left; LUMO, right) of compound 2b.

Figure 3. Frontier molecular orbitals (HOMO, left; LUMO, right) of compound 2c.

**Figure 4.** Frontier molecular orbitals (HOMO, left; LUMO, right) of compound **2d.**

Figure 5. Frontier molecular orbitals (HOMO, left; LUMO, right) of compound 2e.

Figure 6. Frontier molecular orbitals (HOMO, left; LUMO, right) of compound 2f.

Figure 7. Frontier molecular orbitals (HOMO, left; LUMO, right) of compound 2g.

Figure 8. Frontier molecular orbitals (HOMO, left; LUMO, right) of compound 2h.

Figure 9. Frontier molecular orbitals (HOMO, left; LUMO, right) of compound 2i.

Figure 10. Frontier molecular orbitals (HOMO, left; LUMO, right) of compound 2j.

Figure 11. Frontier molecular orbitals (HOMO, left; LUMO, right) of compound 2k.

Figure 12. Frontier molecular orbitals (HOMO, left; LUMO, right) of compound 2l.

Figure 13. Atom numbering of compound 2a used for Hirshfeld charge and condensed Fukui function analyses.

Table 1. Hirshfeld atomic charges and condensed Fukui functions (f⁺, f⁻, f⁰) for compound 2a.ᵃ

| Atom  Number | Hirshfeld Atomic Charges | f^+^ | f^-^ | f^0^ |
| --- | --- | --- | --- | --- |
| 1 | -0.523445 | 0.004137 | 0.002125 | 0.003131 |
| 2 | 0.104953 | 0.002953 | 0.001411 | 0.002182 |
| 3 | -0.517884 | 0.003948 | 0.001945 | 0.002946 |
| 4 | 0.106586 | 0.020377 | 0.010353 | 0.015365 |
| 5 | -0.168016 | 0.028707 | 0.016157 | 0.022432 |
| 6 | 0.072560 | 0.062089 | 0.025586 | 0.043838 |
| 7 | -0.007601 | 0.049274 | 0.020866 | 0.035070 |
| 8 | 0.024573 | 0.084903 | 0.038667 | 0.061785 |
| 9 | -0.034099 | 0.047321 | 0.013851 | 0.030586 |
| 10 | 0.097875 | 0.201725 | 0.091140 | 0.146433 |
| 11 | -0.117104 | 0.123585 | 0.028273 | 0.075929 |
| 12 | 0.117633 | 0.045386 | 0.158343 | 0.101865 |
| 13 | 0.079870 | 0.023978 | 0.030711 | 0.027345 |
| 14 | -0.231578 | 0.030671 | 0.075059 | 0.052865 |
| 15 | 0.007061 | 0.011291 | 0.059536 | 0.035413 |
| 16 | -0.019013 | 0.001582 | 0.006292 | 0.003937 |
| 17 | -0.019421 | 0.006094 | 0.028224 | 0.017159 |
| 18 | 0.003764 | 0.003237 | 0.015656 | 0.009446 |
| 19 | 0.002964 | 0.004745 | 0.027790 | 0.016267 |
| 20 | 0.004833 | 0.003366 | 0.014921 | 0.009143 |
| 21 | 0.004903 | 0.005197 | 0.025536 | 0.015366 |
| 22 | -0.025576 | 0.035427 | 0.143779 | 0.089603 |
| 23 | 0.037110 | 0.046703 | 0.109391 | 0.078047 |
| 24 | 0.006599 | 0.105537 | 0.033214 | 0.069376 |
| 25 | -0.007535 | 0.047773 | 0.021174 | 0.034474 |

*ᵃ Hirshfeld atomic charges were calculated with hydrogen charges summed into the corresponding heavy atoms.*

Figure 14. Atom numbering of compound 2b used for Hirshfeld charge and condensed Fukui function analyses.

Table 2. Hirshfeld atomic charges and condensed Fukui functions (f⁺, f⁻, f⁰) for compound 2b.*ᵃ*

| Atom  Number | Hirshfeld Atomic Charges | f^+^ | f^-^ | f^0^ |
| --- | --- | --- | --- | --- |
| 1 | 0.043961 | 0.002622 | 0.016981 | 0.009802 |
| 2 | 0.000511 | 0.002564 | 0.018312 | 0.010438 |
| 3 | -0.007265 | 0.002990 | 0.016815 | 0.009902 |
| 4 | -0.022065 | 0.006095 | 0.031362 | 0.018729 |
| 5 | -0.026527 | 0.001541 | 0.007591 | 0.004566 |
| 6 | 0.006521 | 0.011168 | 0.062654 | 0.036911 |
| 7 | -0.029670 | 0.035029 | 0.150912 | 0.092971 |
| 8 | 0.034432 | 0.046307 | 0.112544 | 0.079426 |
| 9 | 0.079298 | 0.023736 | 0.032165 | 0.027951 |
| 10 | 0.117186 | 0.045457 | 0.152221 | 0.098839 |
| 11 | -0.117249 | 0.123466 | 0.025239 | 0.074353 |
| 12 | 0.097595 | 0.201492 | 0.087384 | 0.144438 |
| 13 | -0.034201 | 0.047671 | 0.012369 | 0.030020 |
| 14 | 0.024647 | 0.083939 | 0.035731 | 0.059835 |
| 15 | -0.015473 | 0.048648 | 0.018544 | 0.033596 |
| 16 | 0.065203 | 0.061776 | 0.023394 | 0.042585 |
| 17 | -0.166556 | 0.028823 | 0.014798 | 0.021810 |
| 18 | 0.112372 | 0.019552 | 0.008952 | 0.014252 |
| 19 | 0.105083 | 0.003164 | 0.001544 | 0.002354 |
| 20 | -0.509293 | 0.004601 | 0.002270 | 0.003435 |
| 21 | -0.522318 | 0.004894 | 0.002479 | 0.003686 |
| 22 | -0.009161 | 0.048351 | 0.019623 | 0.033987 |
| 23 | 0.006398 | 0.107333 | 0.031266 | 0.069300 |
| 24 | -0.233000 | 0.030637 | 0.072325 | 0.051481 |
| 25 | 0.002117 | 0.005008 | 0.027254 | 0.016131 |
| 26 | -0.002551 | 0.003139 | 0.015271 | 0.009205 |

*ᵃ Hirshfeld atomic charges were calculated with hydrogen charges summed into the corresponding heavy atoms.*

Figure 15. Atom numbering of compound 2c used for Hirshfeld charge and condensed Fukui function analyses.

Table 3. Hirshfeld atomic charges and condensed Fukui functions (f⁺, f⁻, f⁰) for compound 2c.*ᵃ*

| Atom  Number | Hirshfeld Atomic Charges | f^+^ | f^-^ | f^0^ |
| --- | --- | --- | --- | --- |
| 1 | 0.159734 | 0.001509 | 0.012817 | 0.007163 |
| 2 | -0.166578 | 0.001426 | 0.013668 | 0.007547 |
| 3 | 0.063982 | 0.002484 | 0.021822 | 0.012153 |
| 4 | -0.013193 | 0.002817 | 0.020677 | 0.011747 |
| 5 | -0.012808 | 0.006153 | 0.037359 | 0.021756 |
| 6 | -0.034232 | 0.001548 | 0.010918 | 0.006233 |
| 7 | 0.006284 | 0.011115 | 0.067533 | 0.039324 |
| 8 | -0.032922 | 0.034790 | 0.161526 | 0.098158 |
| 9 | 0.032545 | 0.046080 | 0.116671 | 0.081376 |
| 10 | 0.079073 | 0.023632 | 0.034288 | 0.028960 |
| 11 | 0.116984 | 0.045519 | 0.139544 | 0.092531 |
| 12 | -0.117058 | 0.123478 | 0.020246 | 0.071862 |
| 13 | 0.097390 | 0.201603 | 0.080155 | 0.140879 |
| 14 | -0.034256 | 0.047751 | 0.009960 | 0.028856 |
| 15 | 0.024426 | 0.084020 | 0.031238 | 0.057629 |
| 16 | -0.015426 | 0.048750 | 0.015884 | 0.032317 |
| 17 | 0.065247 | 0.061810 | 0.020155 | 0.040983 |
| 18 | -0.166343 | 0.028812 | 0.012554 | 0.020683 |
| 19 | 0.112107 | 0.019557 | 0.007654 | 0.013606 |
| 20 | 0.104961 | 0.003161 | 0.001314 | 0.002237 |
| 21 | -0.509311 | 0.004585 | 0.001914 | 0.003250 |
| 22 | -0.522353 | 0.004894 | 0.002121 | 0.003507 |
| 23 | -0.009300 | 0.048369 | 0.016810 | 0.032590 |
| 24 | 0.006099 | 0.107315 | 0.027347 | 0.067331 |
| 25 | -0.232936 | 0.030650 | 0.066302 | 0.048476 |
| 26 | 0.012082 | 0.004919 | 0.030626 | 0.017772 |
| 27 | -0.014181 | 0.003251 | 0.018893 | 0.011072 |

*ᵃ Hirshfeld atomic charges were calculated with hydrogen charges summed into the corresponding heavy atoms.*

Figure 16. Atom numbering of compound 2d used for Hirshfeld charge and condensed Fukui function analyses.

Table 4. Hirshfeld atomic charges and condensed Fukui functions (f⁺, f⁻, f⁰) for compound 2d.*ᵃ*

| Atom  Number | Hirshfeld Atomic Charges | f+ | f- | f0 |
| --- | --- | --- | --- | --- |
| 1 | -0.517674 | 0.003933 | 0.002025 | 0.002979 |
| 2 | 0.104922 | 0.002939 | 0.001468 | 0.002203 |
| 3 | -0.523790 | 0.004124 | 0.002213 | 0.003169 |
| 4 | 0.106819 | 0.020363 | 0.010816 | 0.015590 |
| 5 | -0.167759 | 0.028577 | 0.016862 | 0.022720 |
| 6 | 0.072723 | 0.061857 | 0.026545 | 0.044201 |
| 7 | -0.007525 | 0.049064 | 0.021798 | 0.035431 |
| 8 | 0.024750 | 0.084871 | 0.039903 | 0.062387 |
| 9 | -0.034089 | 0.046992 | 0.014722 | 0.030857 |
| 10 | 0.098809 | 0.202050 | 0.092118 | 0.147084 |
| 11 | -0.116715 | 0.122987 | 0.029673 | 0.076330 |
| 12 | 0.118963 | 0.044970 | 0.160006 | 0.102488 |
| 13 | 0.080996 | 0.024375 | 0.029595 | 0.026985 |
| 14 | -0.229207 | 0.030675 | 0.075389 | 0.053032 |
| 15 | 0.007810 | 0.011546 | 0.057956 | 0.034751 |
| 16 | -0.013380 | 0.001687 | 0.006076 | 0.003882 |
| 17 | 0.000378 | 0.006339 | 0.028081 | 0.017210 |
| 18 | 0.013272 | 0.003165 | 0.014191 | 0.008678 |
| 19 | 0.016166 | 0.002484 | 0.014392 | 0.008438 |
| 20 | -0.094936 | 0.002894 | 0.015615 | 0.009254 |
| 21 | 0.014133 | 0.003304 | 0.013516 | 0.008410 |
| 22 | 0.026901 | 0.005352 | 0.024202 | 0.014777 |
| 23 | -0.021036 | 0.035783 | 0.139071 | 0.087427 |
| 24 | 0.040521 | 0.047141 | 0.107578 | 0.077360 |
| 25 | 0.006538 | 0.104989 | 0.034191 | 0.069590 |
| 26 | -0.007591 | 0.047545 | 0.021999 | 0.034772 |

*ᵃ Hirshfeld atomic charges were calculated with hydrogen charges summed into the corresponding heavy atoms.*

Figure 17. Atom numbering of compound 2e used for Hirshfeld charge and condensed Fukui function analyses.

Table 5. Hirshfeld atomic charges and condensed Fukui functions (f⁺, f⁻, f⁰) for compound 2e.*ᵃ*

| Atom  Number | Hirshfeld Atomic Charges | f^+^ | f^-^ | f^0^ |
| --- | --- | --- | --- | --- |
| 1 | -0.523522 | 0.000095 | 0.002595 | 0.001345 |
| 2 | 0.104918 | 0.000041 | 0.001727 | 0.000884 |
| 3 | -0.517681 | 0.000050 | 0.002388 | 0.001219 |
| 4 | 0.106893 | 0.000255 | 0.012761 | 0.006508 |
| 5 | -0.167723 | 0.000369 | 0.020148 | 0.010258 |
| 6 | 0.073195 | 0.000658 | 0.030895 | 0.015777 |
| 7 | -0.007161 | 0.000555 | 0.025635 | 0.013095 |
| 8 | 0.025655 | 0.001010 | 0.045279 | 0.023144 |
| 9 | -0.033965 | 0.000141 | 0.018793 | 0.009467 |
| 10 | 0.101320 | 0.003506 | 0.096281 | 0.049893 |
| 11 | -0.116648 | 0.000099 | 0.036442 | 0.018270 |
| 12 | 0.121918 | 0.004405 | 0.166535 | 0.085470 |
| 13 | 0.084007 | 0.004837 | 0.025786 | 0.015312 |
| 14 | -0.225102 | 0.005394 | 0.078466 | 0.041930 |
| 15 | 0.009648 | 0.006168 | 0.050342 | 0.028255 |
| 16 | 0.015245 | 0.052269 | 0.003076 | 0.027672 |
| 17 | 0.012202 | 0.036828 | 0.023841 | 0.030335 |
| 18 | 0.043447 | 0.087920 | 0.010526 | 0.049223 |
| 19 | 0.018063 | 0.022997 | 0.012437 | 0.017717 |
| 20 | 0.249738 | 0.149641 | 0.004295 | 0.076968 |
| 21 | -0.253849 | 0.220297 | 0.009894 | 0.115095 |
| 22 | -0.253383 | 0.220387 | 0.009795 | 0.115091 |
| 23 | 0.042028 | 0.083045 | 0.011671 | 0.047358 |
| 24 | 0.039423 | 0.036600 | 0.020046 | 0.028323 |
| 25 | -0.002106 | 0.035968 | 0.118088 | 0.077028 |
| 26 | 0.052922 | 0.025035 | 0.097665 | 0.061350 |
| 27 | 0.007557 | 0.000889 | 0.038720 | 0.019805 |
| 28 | -0.007053 | 0.000541 | 0.025875 | 0.013208 |

*ᵃ Hirshfeld atomic charges were calculated with hydrogen charges summed into the corresponding heavy atoms.*

Figure 18. Atom numbering of compound 2f used for Hirshfeld charge and condensed Fukui function analyses.

Table 6. Hirshfeld atomic charges and condensed Fukui functions (f⁺, f⁻, f⁰) for compound 2f.*ᵃ*

| Atom  Number | Hirshfeld Atomic Charges | f^+^ | f^-^ | f^0^ |
| --- | --- | --- | --- | --- |
| 1 | -0.293069 | 0.119513 | 0.011671 | 0.065592 |
| 2 | 0.080155 | 0.079578 | 0.005992 | 0.042785 |
| 3 | -0.000150 | 0.087600 | 0.013636 | 0.050618 |
| 4 | 0.052982 | 0.113479 | 0.011593 | 0.062536 |
| 5 | 0.004936 | 0.084355 | 0.024542 | 0.054448 |
| 6 | 0.005648 | 0.093707 | 0.003941 | 0.048824 |
| 7 | 0.008847 | 0.027537 | 0.052235 | 0.039886 |
| 8 | -0.010042 | 0.088992 | 0.124874 | 0.106933 |
| 9 | 0.047573 | 0.059408 | 0.100610 | 0.080009 |
| 10 | 0.082607 | 0.011454 | 0.026663 | 0.019058 |
| 11 | 0.120580 | 0.009969 | 0.165460 | 0.087715 |
| 12 | -0.116747 | 0.000500 | 0.034751 | 0.017626 |
| 13 | 0.100208 | 0.008565 | 0.095849 | 0.052207 |
| 14 | -0.034129 | 0.000403 | 0.017864 | 0.009133 |
| 15 | 0.025305 | 0.002426 | 0.043732 | 0.023079 |
| 16 | -0.015776 | 0.001169 | 0.023831 | 0.012500 |
| 17 | 0.065574 | 0.001478 | 0.029557 | 0.015518 |
| 18 | -0.166201 | 0.000827 | 0.019375 | 0.010101 |
| 19 | 0.112270 | 0.000582 | 0.011542 | 0.006062 |
| 20 | 0.105163 | 0.000101 | 0.001989 | 0.001045 |
| 21 | -0.508827 | 0.000083 | 0.003003 | 0.001543 |
| 22 | -0.522070 | 0.000205 | 0.003235 | 0.001720 |
| 23 | -0.008777 | 0.001231 | 0.025157 | 0.013194 |
| 24 | 0.006928 | 0.002324 | 0.038129 | 0.020227 |
| 25 | -0.226989 | 0.013988 | 0.077797 | 0.045892 |
| 26 | 0.031479 | 0.092354 | 0.020934 | 0.056644 |
| 27 | 0.052503 | 0.098188 | 0.012042 | 0.055115 |

*ᵃ Hirshfeld atomic charges were calculated with hydrogen charges summed into the corresponding heavy atoms.*

Figure 19. Atom numbering of compound 2g used for Hirshfeld charge and condensed Fukui function analyses.

Table 7. Hirshfeld atomic charges and condensed Fukui functions (f⁺, f⁻, f⁰) for compound 2g.*ᵃ*

| Atom  Number | Hirshfeld Atomic Charges | f^+^ | f^-^ | f^0^ |
| --- | --- | --- | --- | --- |
| 1 | -0.523757 | 0.001798 | 0.002425 | 0.002112 |
| 2 | 0.104910 | 0.001237 | 0.001611 | 0.001424 |
| 3 | -0.517650 | 0.001649 | 0.002220 | 0.001935 |
| 4 | 0.106837 | 0.008609 | 0.011859 | 0.010234 |
| 5 | -0.167900 | 0.012264 | 0.018627 | 0.015446 |
| 6 | 0.072893 | 0.025591 | 0.028934 | 0.027263 |
| 7 | -0.007332 | 0.018652 | 0.023800 | 0.021226 |
| 8 | 0.025223 | 0.038019 | 0.042949 | 0.040484 |
| 9 | -0.033970 | 0.014991 | 0.016834 | 0.015913 |
| 10 | 0.099428 | 0.107858 | 0.095339 | 0.101599 |
| 11 | -0.117295 | 0.046642 | 0.033587 | 0.040115 |
| 12 | 0.119368 | 0.022613 | 0.165323 | 0.093968 |
| 13 | 0.081762 | 0.046732 | 0.027807 | 0.037270 |
| 14 | -0.229069 | 0.025940 | 0.078149 | 0.052045 |
| 15 | 0.008613 | 0.051299 | 0.053834 | 0.052567 |
| 16 | -0.001559 | 0.031228 | 0.005060 | 0.018144 |
| 17 | 0.000535 | 0.044546 | 0.025761 | 0.035154 |
| 18 | 0.024637 | 0.038635 | 0.012442 | 0.025538 |
| 19 | -0.039301 | 0.034711 | 0.013312 | 0.024011 |
| 20 | 0.285809 | 0.009992 | 0.003189 | 0.006590 |
| 21 | -0.112692 | 0.006590 | 0.002443 | 0.004517 |
| 22 | -0.129801 | 0.008765 | 0.002946 | 0.005855 |
| 23 | -0.134843 | 0.009314 | 0.003164 | 0.006239 |
| 24 | 0.029042 | 0.029082 | 0.012469 | 0.020776 |
| 25 | 0.026610 | 0.054782 | 0.021750 | 0.038266 |
| 26 | -0.014743 | 0.128427 | 0.129960 | 0.129194 |
| 27 | 0.044130 | 0.117243 | 0.103200 | 0.110222 |
| 28 | 0.007310 | 0.043352 | 0.036862 | 0.040107 |
| 29 | -0.007172 | 0.019431 | 0.024142 | 0.021787 |

*ᵃ Hirshfeld atomic charges were calculated with hydrogen charges summed into the corresponding heavy atoms.*

Figure 20. Atom numbering of compound 2h used for Hirshfeld charge and condensed Fukui function analyses.

Table 8. Hirshfeld atomic charges and condensed Fukui functions (f⁺, f⁻, f⁰) for compound 2h.*ᵃ*

| Atom  Number | Hirshfeld Atomic Charges | f^+^ | f^-^ | f^0^ |
| --- | --- | --- | --- | --- |
| 1 | -0.523574 | 0.004134 | 0.002355 | 0.003244 |
| 2 | 0.105002 | 0.002937 | 0.001557 | 0.002247 |
| 3 | -0.517529 | 0.003921 | 0.002145 | 0.003033 |
| 4 | 0.106546 | 0.020284 | 0.011464 | 0.015874 |
| 5 | -0.167886 | 0.028626 | 0.018006 | 0.023316 |
| 6 | 0.072650 | 0.061853 | 0.028102 | 0.044978 |
| 7 | -0.007523 | 0.048931 | 0.023069 | 0.036000 |
| 8 | 0.024847 | 0.084805 | 0.041961 | 0.063383 |
| 9 | -0.034078 | 0.047060 | 0.016085 | 0.031572 |
| 10 | 0.099147 | 0.202065 | 0.094680 | 0.148372 |
| 11 | -0.116807 | 0.122975 | 0.032111 | 0.077543 |
| 12 | 0.119179 | 0.044793 | 0.163766 | 0.104280 |
| 13 | 0.081266 | 0.024473 | 0.028545 | 0.026509 |
| 14 | -0.230485 | 0.030776 | 0.077476 | 0.054126 |
| 15 | 0.008668 | 0.011527 | 0.055152 | 0.033340 |
| 16 | -0.007589 | 0.001660 | 0.005345 | 0.003502 |
| 17 | -0.006727 | 0.006216 | 0.025663 | 0.015939 |
| 18 | 0.027092 | 0.003336 | 0.013775 | 0.008556 |
| 19 | 0.011549 | 0.004670 | 0.023435 | 0.014052 |
| 20 | 0.018312 | 0.001530 | 0.005866 | 0.003698 |
| 21 | -0.095130 | 0.002294 | 0.009022 | 0.005658 |
| 22 | 0.010222 | 0.005112 | 0.021687 | 0.013400 |
| 23 | -0.018430 | 0.036061 | 0.134265 | 0.085163 |
| 24 | 0.042043 | 0.047440 | 0.105167 | 0.076304 |
| 25 | 0.006803 | 0.104944 | 0.035911 | 0.070428 |
| 26 | -0.007523 | 0.047574 | 0.023390 | 0.035482 |

*ᵃ Hirshfeld atomic charges were calculated with hydrogen charges summed into the corresponding heavy atoms.*

Figure 21. Atom numbering of compound 2i used for Hirshfeld charge and condensed Fukui function analyses.

Table 9. Hirshfeld atomic charges and condensed Fukui functions (f⁺, f⁻, f⁰) for compound 2i.*ᵃ*

| Atom  Number | Hirshfeld Atomic Charges | f^+^ | f^-^ | f^0^ |
| --- | --- | --- | --- | --- |
| 1 | -0.517504 | 0.004005 | 0.002275 | 0.003140 |
| 2 | 0.104826 | 0.002940 | 0.001612 | 0.002276 |
| 3 | -0.524179 | 0.004006 | 0.002413 | 0.003210 |
| 4 | 0.107372 | 0.020911 | 0.011824 | 0.016367 |
| 5 | -0.168106 | 0.027567 | 0.018788 | 0.023178 |
| 6 | 0.072797 | 0.061207 | 0.029083 | 0.045145 |
| 7 | -0.004310 | 0.047783 | 0.023435 | 0.035609 |
| 8 | 0.008287 | 0.120315 | 0.041434 | 0.080874 |
| 9 | -0.034146 | 0.048059 | 0.017003 | 0.032531 |
| 10 | 0.099885 | 0.199263 | 0.095480 | 0.147372 |
| 11 | -0.116705 | 0.121037 | 0.033392 | 0.077214 |
| 12 | 0.120322 | 0.044012 | 0.164397 | 0.104204 |
| 13 | 0.082195 | 0.023758 | 0.027577 | 0.025668 |
| 14 | -0.228202 | 0.029865 | 0.077371 | 0.053618 |
| 15 | 0.009457 | 0.011313 | 0.053767 | 0.032540 |
| 16 | -0.003508 | 0.001687 | 0.005257 | 0.003472 |
| 17 | 0.010467 | 0.006137 | 0.025090 | 0.015614 |
| 18 | 0.029013 | 0.003108 | 0.012433 | 0.007770 |
| 19 | 0.012097 | 0.002360 | 0.012285 | 0.007323 |
| 20 | -0.071715 | 0.002812 | 0.013630 | 0.008221 |
| 21 | 0.014830 | 0.001443 | 0.005274 | 0.003359 |
| 22 | -0.071849 | 0.002218 | 0.008563 | 0.005390 |
| 23 | 0.024969 | 0.005070 | 0.020971 | 0.013020 |
| 24 | -0.014431 | 0.035106 | 0.130220 | 0.082663 |
| 25 | 0.044868 | 0.046305 | 0.103339 | 0.074822 |
| 26 | 0.024028 | 0.072858 | 0.038300 | 0.055579 |
| 27 | -0.010727 | 0.054858 | 0.024783 | 0.039820 |

*ᵃ Hirshfeld atomic charges were calculated with hydrogen charges summed into the corresponding heavy atoms.*

Figure 22. Atom numbering of compound 2j used for Hirshfeld charge and condensed Fukui function analyses.

Table 10. Hirshfeld atomic charges and condensed Fukui functions (f⁺, f⁻, f⁰) for compound 2j.*ᵃ*

| Atom  Number | Hirshfeld Atomic Charges | f^+^ | f^-^ | f^0^ |
| --- | --- | --- | --- | --- |
| 1 | -0.524083 | 0.004147 | 0.002036 | 0.003092 |
| 2 | 0.104705 | 0.002960 | 0.001347 | 0.002154 |
| 3 | -0.517554 | 0.003958 | 0.001849 | 0.002904 |
| 4 | 0.107162 | 0.020616 | 0.009968 | 0.015292 |
| 5 | -0.167560 | 0.028747 | 0.015414 | 0.022081 |
| 6 | 0.072605 | 0.062419 | 0.024436 | 0.043428 |
| 7 | -0.007889 | 0.049421 | 0.020055 | 0.034738 |
| 8 | 0.023989 | 0.085735 | 0.036955 | 0.061345 |
| 9 | -0.034350 | 0.047806 | 0.013325 | 0.030565 |
| 10 | 0.096861 | 0.203619 | 0.086672 | 0.145146 |
| 11 | -0.126543 | 0.131886 | 0.030948 | 0.081417 |
| 12 | 0.111506 | 0.044758 | 0.147470 | 0.096114 |
| 13 | 0.080217 | 0.025825 | 0.029851 | 0.027838 |
| 14 | -0.233242 | 0.032419 | 0.068002 | 0.050210 |
| 15 | 0.009226 | 0.009428 | 0.062578 | 0.036003 |
| 16 | -0.018905 | 0.001448 | 0.006767 | 0.004108 |
| 17 | -0.015064 | 0.006731 | 0.030290 | 0.018510 |
| 18 | 0.005372 | 0.002268 | 0.014729 | 0.008498 |
| 19 | -0.009991 | 0.002156 | 0.017970 | 0.010063 |
| 20 | -0.010976 | 0.000117 | 0.001280 | 0.000698 |
| 21 | 0.002278 | 0.000622 | 0.006116 | 0.003369 |
| 22 | 0.004003 | 0.000497 | 0.003649 | 0.002073 |
| 23 | 0.001896 | 0.000964 | 0.006729 | 0.003846 |
| 24 | 0.003733 | 0.000594 | 0.003698 | 0.002146 |
| 25 | 0.002263 | 0.000781 | 0.006154 | 0.003468 |
| 26 | 0.006041 | 0.002734 | 0.013878 | 0.008306 |
| 27 | 0.008805 | 0.004249 | 0.027614 | 0.015932 |
| 28 | -0.017610 | 0.026525 | 0.141739 | 0.084132 |
| 29 | 0.045658 | 0.042839 | 0.116487 | 0.079663 |
| 30 | 0.005376 | 0.106042 | 0.031879 | 0.068961 |
| 31 | -0.008016 | 0.047716 | 0.020121 | 0.033918 |

*ᵃ Hirshfeld atomic charges were calculated with hydrogen charges summed into the corresponding heavy atoms.*

Figure 23. Atom numbering of compound 2k used for Hirshfeld charge and condensed Fukui function analyses.

Table 11. Hirshfeld atomic charges and condensed Fukui functions (f⁺, f⁻, f⁰) for compound 2k.*ᵃ*

| Atom  Number | Hirshfeld Atomic Charges | f^+^ | f^-^ | f^0^ |
| --- | --- | --- | --- | --- |
| 1 | -0.523706 | 0.004128 | 0.002134 | 0.003131 |
| 2 | 0.104914 | 0.002941 | 0.001414 | 0.002177 |
| 3 | -0.517828 | 0.003945 | 0.001953 | 0.002949 |
| 4 | 0.106997 | 0.020383 | 0.010410 | 0.015396 |
| 5 | -0.167794 | 0.028614 | 0.016203 | 0.022408 |
| 6 | 0.072571 | 0.061971 | 0.025656 | 0.043814 |
| 7 | -0.007655 | 0.049202 | 0.021004 | 0.035103 |
| 8 | 0.024486 | 0.084922 | 0.038769 | 0.061846 |
| 9 | -0.034181 | 0.047191 | 0.013937 | 0.030564 |
| 10 | 0.098241 | 0.202089 | 0.091093 | 0.146591 |
| 11 | -0.116604 | 0.123179 | 0.028203 | 0.075691 |
| 12 | 0.118453 | 0.045259 | 0.158225 | 0.101742 |
| 13 | 0.080255 | 0.024205 | 0.030592 | 0.027398 |
| 14 | -0.231452 | 0.030809 | 0.074877 | 0.052843 |
| 15 | 0.007663 | 0.011381 | 0.059465 | 0.035423 |
| 16 | -0.020622 | 0.001718 | 0.006992 | 0.004355 |
| 17 | 0.001147 | 0.006204 | 0.028627 | 0.017416 |
| 18 | 0.012103 | 0.003260 | 0.015992 | 0.009626 |
| 19 | 0.083748 | 0.002717 | 0.016424 | 0.009570 |
| 20 | -0.140472 | 0.001591 | 0.009192 | 0.005391 |
| 21 | 0.013417 | 0.003401 | 0.014946 | 0.009174 |
| 22 | 0.025650 | 0.005316 | 0.025486 | 0.015401 |
| 23 | -0.025367 | 0.035677 | 0.144208 | 0.089943 |
| 24 | 0.037528 | 0.047018 | 0.109760 | 0.078389 |
| 25 | 0.006266 | 0.105237 | 0.033224 | 0.069230 |
| 26 | -0.007758 | 0.047650 | 0.021217 | 0.034434 |

*ᵃ Hirshfeld atomic charges were calculated with hydrogen charges summed into the corresponding heavy atoms.*

Figure 24. Atom numbering of compound 2l used for Hirshfeld charge and condensed Fukui function analyses.

Table 12. Hirshfeld atomic charges and condensed Fukui functions (f⁺, f⁻, f⁰) for compound 2l.*ᵃ*

| Atom  Number | Hirshfeld Atomic Charges | f^+^ | f^-^ | f^0^ |
| --- | --- | --- | --- | --- |
| 1 | -0.517851 | 0.004027 | 0.002144 | 0.003086 |
| 2 | 0.104846 | 0.002959 | 0.001519 | 0.002239 |
| 3 | -0.523870 | 0.004041 | 0.002279 | 0.003160 |
| 4 | 0.107294 | 0.021091 | 0.011137 | 0.016114 |
| 5 | -0.168221 | 0.027694 | 0.017599 | 0.022646 |
| 6 | 0.072449 | 0.061665 | 0.027556 | 0.044610 |
| 7 | -0.004765 | 0.048243 | 0.022137 | 0.035190 |
| 8 | 0.007669 | 0.121895 | 0.039824 | 0.080860 |
| 9 | -0.034210 | 0.048905 | 0.015495 | 0.032200 |
| 10 | 0.097731 | 0.198933 | 0.094903 | 0.146918 |
| 11 | -0.116966 | 0.121944 | 0.031079 | 0.076511 |
| 12 | 0.117142 | 0.045252 | 0.163352 | 0.104302 |
| 13 | 0.078980 | 0.022704 | 0.029571 | 0.026138 |
| 14 | -0.239666 | 0.030264 | 0.078790 | 0.054527 |
| 15 | 0.013165 | 0.010071 | 0.052289 | 0.031180 |
| 16 | -0.015550 | 0.001012 | 0.003641 | 0.002326 |
| 17 | -0.008679 | 0.005957 | 0.026802 | 0.016380 |
| 18 | 0.006503 | 0.002284 | 0.011007 | 0.006645 |
| 19 | 0.009808 | 0.004036 | 0.021708 | 0.012872 |
| 20 | -0.013629 | 0.000978 | 0.004369 | 0.002674 |
| 21 | 0.007247 | 0.001877 | 0.009363 | 0.005620 |
| 22 | 0.004870 | 0.001634 | 0.007714 | 0.004674 |
| 23 | 0.004772 | 0.002164 | 0.011655 | 0.006909 |
| 24 | -0.009413 | 0.002176 | 0.010429 | 0.006302 |
| 25 | -0.011435 | 0.001230 | 0.005567 | 0.003398 |
| 26 | -0.019184 | 0.032949 | 0.132420 | 0.082684 |
| 27 | 0.038615 | 0.045310 | 0.105626 | 0.075468 |
| 28 | 0.023342 | 0.073006 | 0.036697 | 0.054851 |
| 29 | -0.011027 | 0.055709 | 0.023327 | 0.039518 |

*ᵃ Hirshfeld atomic charges were calculated with hydrogen charges summed into the corresponding heavy atoms.*
