# Supplementary material for: Investigation of the In Vitro and In Silico Anticancer Potential of Novel 4‐Arylthiazole‐Hydrazone Derivatives
Source: Chem Biol Drug Des. 2026 Aug 30;108(3):e70387. doi: 10.1111/cbdd.70387 (PMC13527178; doi:10.1111/cbdd.70387)
Supplement: Supplementary file 2 [file CBDD-108-e70387-s002.docx]

**Investigation of the *in vitro* and *in silico* anticancer potential of novel 4-arylthiazole-hydrazone derivatives**

Zeynep Zişan Demirci^1,*^, Erol Akgün^1,2^, Gülşen Akalın Çiftçi^3^, Leyla Yurttaş^4,*^

[Figure 1. IR spectrum of compound **2a**	2](#_Toc223172130)

[Figure 2. ^1^ H-NMR spectrum of compound **2a**	2](#_Toc223172131)

[Figure 3. ^13^ C-NMR spectrum of compound **2a**	3](#_Toc223172132)

[Figure 4. LC-MS spectrum of compound **2a**	3](#_Toc223172133)

[Figure 5. IR spectrum of compound **2b**	4](#_Toc223172134)

[Figure 6. ^1^ H-NMR spectrum of compound **2b**	4](#_Toc223172135)

[Figure 7. ^13^ C-NMR spectrum of compound **2b**	5](#_Toc223172136)

[Figure 8. LC-MS spectrum of compound **2b**	5](#_Toc223172137)

[Figure 9. IR spectrum of compound **2c**	6](#_Toc223172138)

[Figure 10. ^1^ H-NMR spectrum of compound **2c**	6](#_Toc223172139)

[Figure 11. ^13^ C-NMR spectrum of compound **2c**	7](#_Toc223172140)

[Figure 12. LC-MS spectrum of compound **2c**	7](#_Toc223172141)

[Figure 13. IR spectrum of compound **2d**	8](#_Toc223172142)

[Figure 14. ^1^ H-NMR spectrum of compound **2d**	8](#_Toc223172143)

[Figure 15. ^13^ C-NMR spectrum of compound **2d**	9](#_Toc223172144)

[Figure 16. LC-MS spectrum of compound **2d**	9](#_Toc223172145)

[Figure 17. IR spectrum of compound **2e**	10](#_Toc223172146)

[Figure 18. ^1^ H-NMR spectrum of compound **2e**	10](#_Toc223172147)

[Figure 19. ^13^ C-NMR spectrum of compound **2e**	11](#_Toc223172148)

[Figure 20. LC-MS spectrum of compound **2e**	11](#_Toc223172149)

[Figure 21. IR spectrum of compound **2f**	12](#_Toc223172150)

[Figure 22. ^1^ H-NMR spectrum of compound **2f**	12](#_Toc223172151)

[Figure 23. ^13^ C-NMR spectrum of compound **2f**	13](#_Toc223172152)

[Figure 24. LC-MS spectrum of compound **2f**	13](#_Toc223172153)

[Figure 25. IR spectrum of compound **2g**	14](#_Toc223172154)

[Figure 26. ^1^ H-NMR spectrum of compound **2g**	14](#_Toc223172155)

[Figure 27. ^13^ C-NMR spectrum of compound **2g**	15](#_Toc223172156)

[Figure 28. LC-MS spectrum of compound **2g**	15](#_Toc223172157)

[Figure 29. IR spectrum of compound **2h**	16](#_Toc223172158)

[Figure 30. ^1^ H-NMR spectrum of compound **2h**	16](#_Toc223172159)

[Figure 31. ^13^ C-NMR spectrum of compound **2h**	17](#_Toc223172160)

[Figure 32. LC-MS spectrum of compound **2h**	17](#_Toc223172161)

[Figure 33. IR spectrum of compound **2i**	18](#_Toc223172162)

[Figure 34. ^1^ H-NMR spectrum of compound **2i**	18](#_Toc223172163)

[Figure 35. ^13^ C-NMR spectrum of compound **2i**	19](#_Toc223172164)

[Figure 36. LC-MS spectrum of compound **2i**	19](#_Toc223172165)

[Figure 37. IR spectrum of compound **2j**	20](#_Toc223172166)

[Figure 38. ^1^ H-NMR spectrum of compound **2j**	20](#_Toc223172167)

[Figure 39. ^13^ C-NMR spectrum of compound **2j**	21](#_Toc223172168)

[Figure 40. LC-MS spectrum of compound **2j**	21](#_Toc223172169)

[Figure 41. IR spectrum of compound **2k**	22](#_Toc223172170)

[Figure 42. ^1^ H-NMR spectrum of compound **2k**	22](#_Toc223172171)

[Figure 43. ^13^ C-NMR spectrum of compound **2k**	23](#_Toc223172172)

[Figure 44. LC-MS spectrum of compound **2k**	23](#_Toc223172173)

[Figure 45. IR spectrum of compound **2l**	24](#_Toc223172174)

[Figure 46. ^1^ H-NMR spectrum of compound **2l**	24](#_Toc223172175)

[Figure 47. ^13^ C-NMR spectrum of compound **2l**	25](#_Toc223172176)

[Figure 48. LC-MS spectrum of compound **2l**	25](#_Toc223172169)

[Figure 49. Dose-response graphs of the compounds against A549 cell line	26](#_Toc223172177)

[Figure 50. Dose-response graphs of the compounds against MCF-7 cell line	28](#_Toc223172175)

[Figure 51. Dose-response graphs of the compounds against L929 cell line](#_Toc223172176)	30

Figure 1. IR spectrum of compound 2a

Figure 2. ^1^ H-NMR spectrum of compound 2a

Figure 3. ^13^ C-NMR spectrum of compound 2a

Figure 4. LC-MS spectrum of compound 2a

Figure 5. IR spectrum of compound 2b

Figure 6. ^1^ H-NMR spectrum of compound 2b

Figure 7. ^13^ C-NMR spectrum of compound 2b

Figure 8. LC-MS spectrum of compound 2b

Figure 9. IR spectrum of compound 2c

Figure 10. ^1^ H-NMR spectrum of compound 2c

Figure 11. ^13^ C-NMR spectrum of compound 2c

Figure 12. LC-MS spectrum of compound 2c

Figure 13. IR spectrum of compound 2d

Figure 14. ^1^ H-NMR spectrum of compound 2d

Figure 15. ^13^ C-NMR spectrum of compound 2d

Figure 16. LC-MS spectrum of compound 2d

Figure 17. IR spectrum of compound 2e

Figure 18. ^1^ H-NMR spectrum of compound 2e

Figure 19. ^13^ C-NMR spectrum of compound 2e

Figure 20. LC-MS spectrum of compound 2e

Figure 21. IR spectrum of compound 2f

Figure 22. ^1^ H-NMR spectrum of compound 2f

Figure 23. ^13^ C-NMR spectrum of compound 2f

Figure 24. LC-MS spectrum of compound 2f

Figure 25. IR spectrum of compound 2g

Figure 26. ^1^ H-NMR spectrum of compound 2g

Figure 27. ^13^ C-NMR spectrum of compound 2g

Figure 28. LC-MS spectrum of compound 2g

Figure 29. IR spectrum of compound 2h

Figure 30. ^1^ H-NMR spectrum of compound 2h

Figure 31. ^13^ C-NMR spectrum of compound 2h

Figure 32. LC-MS spectrum of compound 2h

Figure 33. IR spectrum of compound 2i

Figure 34. ^1^ H-NMR spectrum of compound 2i

Figure 35. ^13^ C-NMR spectrum of compound 2i

Figure 36. LC-MS spectrum of compound 2i

Figure 37. IR spectrum of compound 2j

Figure 38. ^1^ H-NMR spectrum of compound 2j

Figure 39. ^13^ C-NMR spectrum of compound 2j

Figure 40. LC-MS spectrum of compound 2j

Figure 41. IR spectrum of compound 2k

Figure 42. ^1^ H-NMR spectrum of compound 2k

Figure 43. ^13^ C-NMR spectrum of compound 2k

Figure 44. LC-MS spectrum of compound 2k

Figure 45. IR spectrum of compound 2l

Figure 46. ^1^ H-NMR spectrum of compound 2l

Figure 47. ^13^ C-NMR spectrum of compound 2l

Figure 48. LC-MS spectrum of compound 2l

2b

| Concentration (µM) | Viability % |
| --- | --- |
| 1000 | -20.633 |
| 500 | 6.61058 |
| 100 | 79.1466 |
| 50 | 84.996 |
| 10 | 71.0737 |
| 5 | 69.6514 |
| 1 | 74.7396 |

2c

| Concentration (µM) | Viability % |
| --- | --- |
| 1000 | -4.26683 |
| 500 | -1.46234 |
| 100 | 66.6266 |
| 50 | 73.8381 |
| 10 | 67.1474 |
| 5 | 66.9271 |
| 1 | 69.8317 |

2e

| Concentration (µM) | Viability (%) |
| --- | --- |
| 1000 | 0 |
| 500 | -10.1763 |
| 100 | 74.2388 |
| 50 | 81.4103 |
| 10 | 73.2372 |
| 5 | 74.1186 |
| 1 | 74.0785 |

2f

| Concentration (µM) | Viability (%) |
| --- | --- |
| 1000 | -24.9599 |
| 500 | 11.238 |
| 100 | 83.0529 |
| 50 | 75.2804 |
| 10 | 62.8205 |
| 5 | 64.4631 |
| 1 | 68.4495 |

2h

| Concentration (µM | Viability % |
| --- | --- |
| 1000 | -31.2901 |
| 500 | 11.5184 |
| 100 | 95.633 |
| 50 | 92.9487 |
| 10 | 77.7043 |
| 5 | 80.2284 |
| 1 | 83.6338 |

2k

| Concentration (µM) | Viability %) |
| --- | --- |
| 1000 | 11.2981 |
| 500 | 30.4888 |
| 100 | 57.3518 |
| 50 | 77.484 |
| 10 | 79.7676 |
| 5 | 84.5553 |
| 1 | 85.3766 |

2l

| Concentration (µM) | Viability %) |
| --- | --- |
| 1000 | 22.4159 |
| 500 | 48.3974 |
| 100 | 64.7436 |
| 50 | 79.4071 |
| 10 | 79.4671 |
| 5 | 84.6154 |
| 1 | 85.2564 |

*Figure 49. Dose-response graphs of the compounds against A549 cell line*

2c

| Concentrations (µM) | Viability (%) |
| --- | --- |
| 1000 | -35.4545 |
| 500 | 6.7316 |
| 100 | 36.4069 |
| 50 | 59.8918 |
| 10 | 50.1299 |
| 5 | 56.4069 |
| 1 | 53.5281 |

2d

| Concentration (µM) | viability % |
| --- | --- |
| 1000 | 128.723 |
| 500 | 98.7229 |
| 100 | 49.5455 |
| 50 | 51.9048 |
| 10 | 73.5065 |
| 5 | 64.9351 |
| 1 | 71.3203 |

2e

| Concentration (µM) | viability % |
| --- | --- |
| 1000 | -35.5195 |
| 500 | -9.11255 |
| 100 | 66.5801 |
| 50 | 77.619 |
| 10 | 59.632 |
| 5 | 55.974 |
| 1 | 50.7359 |

2f

| Concentration (µM) | viability % |
| --- | --- |
| 1000 | 17.0346 |
| 500 | 18.29 |
| 100 | 65.8009 |
| 50 | 61.1472 |
| 10 | 52.8355 |
| 5 | 55.3896 |
| 1 | 50.8225 |

2g

| Concentration (µM) | Viability % |
| --- | --- |
| 1000 | 89.026 |
| 500 | 78.7229 |
| 100 | 40.0216 |
| 50 | 28.5498 |
| 10 | 40.1948 |
| 5 | 45.7359 |
| 1 | 56.039 |

2h

| Concentration (µM) | Viability % |
| --- | --- |
| 1000 | -60.8225 |
| 500 | -24.8701 |
| 100 | 42.0563 |
| 50 | 45.8009 |
| 10 | 42.5974 |
| 5 | 41.6883 |
| 1 | 51.7749 |

2i

| Concentration (µM) | Viability % |
| --- | --- |
| 1000 | 67.7489 |
| 500 | 61.7532 |
| 100 | 92.1212 |
| 50 | 74.5671 |
| 10 | 52.0346 |
| 5 | 53.1169 |
| 1 | 56.4286 |

2k

| Concentration (µM) | Viability % |
| --- | --- |
| 1000 | 3.44156 |
| 500 | 25.1299 |
| 100 | 25.7359 |
| 50 | 26.9481 |
| 10 | 45 |
| 5 | 46.5368 |
| 1 | 49.5238 |

2l

| Concentration (µM) | Viability % |
| --- | --- |
| 1000 | 26.4935 |
| 500 | 32.8139 |
| 100 | 28.29 |
| 50 | 31.6017 |
| 10 | 46.3203 |
| 5 | 48.2035 |
| 1 | 50.5411 |

Cisplatin

| Concentration | Viability % |
| --- | --- |
| 1000 | -3.50877 |
| 500 | 5.88972 |
| 100 | 23.3396 |
| 50 | 35.213 |
| 10 | 61.4662 |
| 5 | 67.0113 |
| 1 | 71.6792 |

*Figure 50. Dose-response graphs of the compounds against MCF-7 cell line*

2b

| Concentration (µM) | Viability (%) |
| --- | --- |
| 1000 | -25.1246 |
| 500 | -2.3962 |
| 100 | 62.4911 |
| 50 | 75.8482 |
| 10 | 71.0083 |
| 5 | 77.3191 |
| 1 | 86.9276 |

2c

| Concentration (µM) | Viability (%) |
| --- | --- |
| 1000 | -16.2752 |
| 500 | -2.27758 |
| 100 | 72.7165 |
| 50 | 71.1981 |
| 10 | 75.089 |
| 5 | 75.9905 |
| 1 | 86.5006 |

2d

| Concentration (µM) | Viability % |
| --- | --- |
| 1000 | 79.8814 |
| 500 | 64.3416 |
| 100 | 55.255 |
| 50 | 71.8149 |
| 10 | 75.9668 |
| 5 | 80.688 |
| 1 | 93.452 |

2e

| Concentration (µM) | Viability % |
| --- | --- |
| 1000 | -55.9905 |
| 500 | -19.8339 |
| 100 | 91.4828 |
| 50 | 111.673 |
| 10 | 75.4211 |
| 5 | 80.3796 |
| 1 | 87.8529 |

2f

| Concentration (µM) | Viability % |
| --- | --- |
| 1000 | -19.4781 |
| 500 | 19.4069 |
| 100 | 84.8636 |
| 50 | 81.2811 |
| 10 | 70.7948 |
| 5 | 78.2918 |
| 1 | 85.4804 |

2g

| Concentration (µM) | Viability % |
| --- | --- |
| 1000 | 128.019 |
| 500 | 99.739 |
| 100 | 79.4306 |
| 50 | 58.624 |
| 10 | 82.7284 |
| 5 | 87.07 |
| 1 | 93.1673 |

2h

| Concentration (µM) | Viability % |
| --- | --- |
| 1000 | -35.3737 |
| 500 | -6.69039 |
| 100 | 68.707 |
| 50 | 70.3677 |
| 10 | 73.7129 |
| 5 | 73.6655 |
| 1 | 84.3654 |

2i

| Concentration (µM) | Viability % |
| --- | --- |
| 1000 | 17.6157 |
| 500 | 36.5125 |
| 100 | 78.2444 |
| 50 | 75.777 |
| 10 | 73.9265 |
| 5 | 73.879 |
| 1 | 84.0807 |

2k

| Concentration (µM) | Viability (%) |
| --- | --- |
| 1000 | -4.34164 |
| 500 | 8.11388 |
| 100 | 71.6251 |
| 50 | 74.3535 |
| 10 | 73.7129 |
| 5 | 73.452 |
| 1 | 84.223 |

2l

| Concentration (µM) | Viability (%) |
| --- | --- |
| 1000 | 8.82562 |
| 500 | 29.3238 |
| 100 | 80.3796 |
| 50 | 76.6311 |
| 10 | 73.3571 |
| 5 | 73.5231 |
| 1 | 84.0807 |

*Figure 51.* *Dose-response graphs of the compounds against L929 cell line*
